# Distinct and shared multimodal neuroimaging patterns in clinical frontotemporal dementia syndromes

**DOI:** 10.1093/braincomms/fcag341

**Published:** 2026-09-10

**Authors:** Irene Sintini, Neha Singh-Reilly, Farwa Ali, Joseph R Duffy, Heather M Clark, Rene L Utianski, Gabriela F Meade, Mary M Machulda, Ryota Satoh, Dennis W Dickson, Val J Lowe, Keith A Josephs, Jennifer L Whitwell

**Affiliations:** Department of Radiology, Mayo Clinic, Rochester, MN 55905, USA; Department of Radiology, Mayo Clinic, Rochester, MN 55905, USA; Department of Neurology, Mayo Clinic, Rochester, MN 55905, USA; Department of Neurology, Mayo Clinic, Rochester, MN 55905, USA; Department of Neurology, Mayo Clinic, Rochester, MN 55905, USA; Department of Neurology, Mayo Clinic, Rochester, MN 55905, USA; Department of Neurology, Mayo Clinic, Rochester, MN 55905, USA; Department of Psychiatry and Psychology, Mayo Clinic, Rochester, MN 55905, USA; Department of Radiology, Mayo Clinic, Rochester, MN 55905, USA; Department of Neuroscience, Mayo Clinic, Jacksonville, FL 32224, USA; Department of Radiology, Mayo Clinic, Rochester, MN 55905, USA; Department of Neurology, Mayo Clinic, Rochester, MN 55905, USA; Department of Radiology, Mayo Clinic, Rochester, MN 55905, USA

**Keywords:** frontotemporal dementia, MRI, tau-PET, FDG-PET, principal component analysis

## Abstract

Clinical syndromes of frontotemporal dementia present both distinct and overlapping neuroimaging abnormalities and, consequently, domains of impairment. The aim of this study was to determine how well covariance patterns from multiple neuroimaging modalities discriminate across clinical frontotemporal dementia syndromes. Four-hundred participants with a clinical frontotemporal dementia diagnosis, including behavioural variant of frontotemporal dementia, semantic variant of primary progressive aphasia, right temporal variant of frontotemporal dementia, primary progressive aphasia, apraxia of speech with agrammatic aphasia, primary progressive apraxia of speech, progressive supranuclear palsy and corticobasal syndrome, and 109 cognitively unimpaired participants underwent extensive neurological and neuropsychological assessments, structural magnetic resonance imaging (MRI), flortaucipir-PET for tau and [^18^F] fluorodeoxyglucose-PET. Multimodal imaging covariance was investigated at the voxel-level using principal component analysis after adjusting for age and sex effects. Linear regression models were fit to investigate the relationship between principal components and clinical scores. Multinomial logistic regression models were used to investigate the ability of imaging principal components to discriminate across syndromes. The first principal component, which describes the largest source of variability in the data, was a pattern of widespread cortical atrophy (21% variability explained) and of frontoparietal and temporal hypometabolism (21%): scores were lowest in the behavioural variant of frontotemporal dementia and correlated with general cognitive impairment. On tau-PET, the first principal component (43% of variability) did not significantly differ among syndromes and captured a widespread cortical tau uptake, with a focus on the temporal lobe. In all three modalities, the second principal component captured temporal lobe imaging abnormalities, contrasting the motor and semantic clinical syndromes. Subsequent components described smaller variability. In cross-validation, the classifiers based on principal components from MRI (multiclass area under the receiver operating characteristic: 0.76) and [^18^F]fluorodeoxyglucose-PET (0.79) outperformed tau-PET (0.71). In cross-validation, the sensitivity of the image-based classifiers was heterogeneous across syndromes and modalities, with the highest values for progressive supranuclear palsy and semantic variant of primary progressive aphasia and the lowest for primary progressive aphasia and corticobasal syndrome. Our study found that the covariance components of structural and molecular neuroimaging underlying frontotemporal dementia clinical syndromes exist both as a continuum and with syndrome-specific patterns. More than one imaging model was able to correctly classify several frontotemporal dementia clinical syndromes with good specificity and sensitivity, highlighting the value of imaging in aiding patient diagnosis.

## Teaser

Sintini *et al*. report that principal components of structural and molecular neuroimaging in clinical syndromes of frontotemporal dementia exist as a continuum and with syndrome-specific patterns. MRI and [^18^F]fluorodeoxyglucose-PET principal components diagnostic capabilities outperformed tau-PET, with the highest sensitivity for the semantic variant of primary progressive aphasia and progressive supranuclear palsy.

## Graphical Abstract

**Graphical Abstract:**
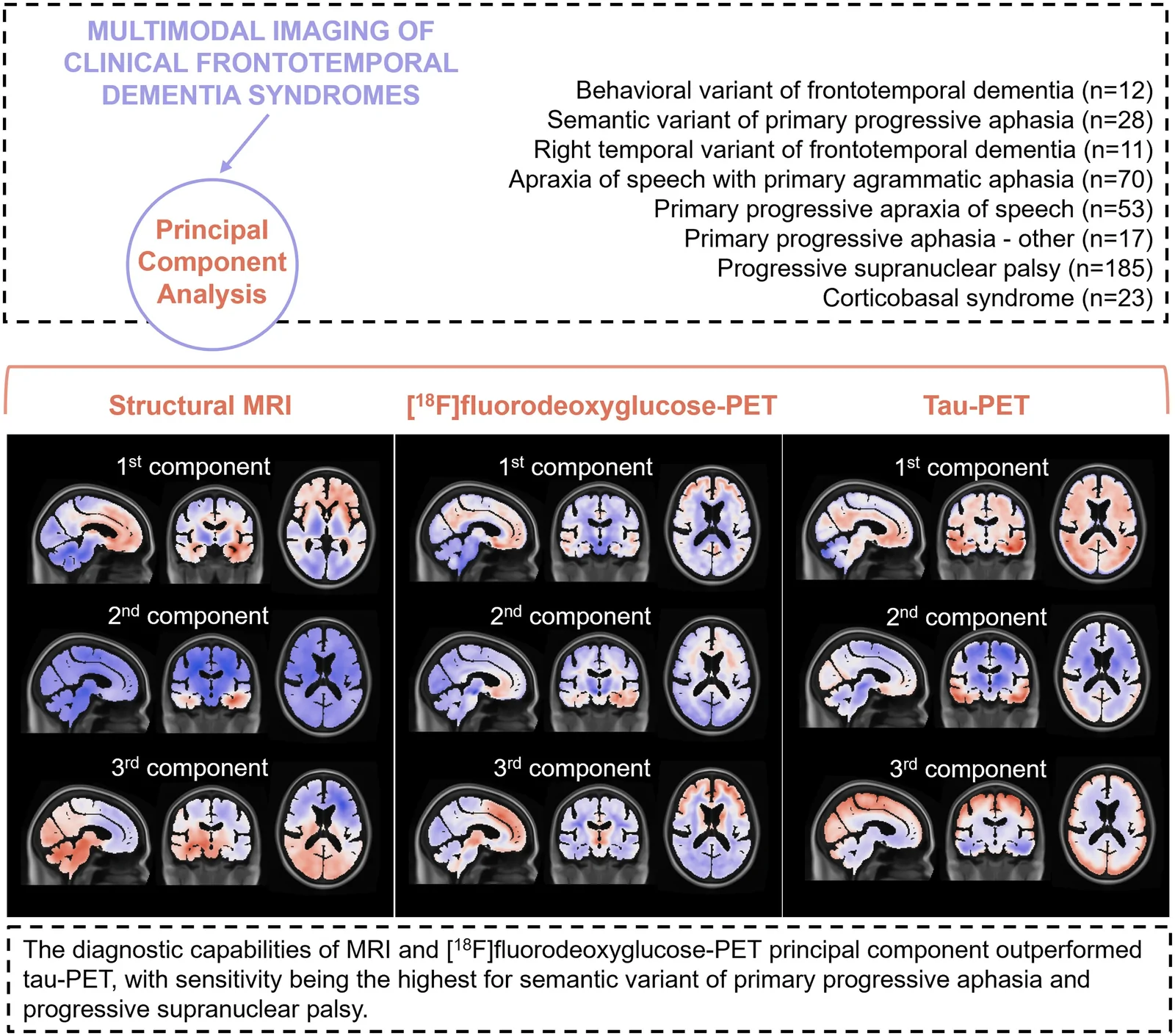

## Introduction

Frontotemporal dementia (FTD) encompasses a spectrum of heterogeneous clinical syndromes that target the frontal and temporal lobes whose domains of impairment span from behaviour and personality to speech, language, and movement.^1,2^ The three classic FTD syndromes that were described in the 1998 diagnostic criteria include the behavioural variant of FTD (bvFTD),^3^ which primarily alters personality and social conduct, semantic variant of primary progressive aphasia (svPPA),^4^ which is characterized by naming and word comprehension impairment and associative agnosia, and progressive non-fluent aphasia, characterized by effortful speech production and grammatical errors.^4^ Since that time, it has been recognized that progressive non-fluent aphasia encompasses patients with agrammatic aphasia and apraxia of speech (AOS). Patients can present with both apraxia of speech and agrammatic aphasia (AOS-PAA),^5^ an isolated primary progressive apraxia of speech in the absence of aphasia (PPAOS)^6^ or an isolated progressive agrammatic aphasia (PAA).^7^ Patients with AOS-PAA and PAA could also be classified as agrammatic variant of primary progressive aphasia.^8^ A variant of FTD with right anterior temporal lobe predominance [also referred to as right temporal variant of FTD (rtvFTD)] has also been recognized, and it is characterized by mental rigidity, behavioural abnormalities, anomia, and facial recognition deficits.^9^ The atypical parkinsonian disorders of progressive supranuclear palsy (PSP)^10^ and corticobasal syndrome (CBS)^11^ are also considered to be FTD syndromes.^12,13^

These different FTD syndromes commonly overlap,^14^ with atypical parkinsonian syndromes often developing in patients with AOS-PAA^15^ and PPAOS,^16^ and clinical overlap observed between bvFTD, svPPA, and rtvFTD.^17^ Frontotemporal lobar degeneration (FTLD) is the most common underlying neuropathological change in patients clinically diagnosed with FTD syndromes. Proteinopathies within FTLD most commonly include FTLD-tau, with either 3-repeat (3R) or 4-repeat (4R) tauopathies, and FTLD with TAR DNA-binding protein 43 (TDP-43).^2^ The correspondence between clinical FTD and neuropathological FTLD syndromes remains far from perfect.^18-21^ Hence, neuroimaging may help close the gap between the clinic and neuropathology, being a powerful tool for discriminating *in vivo* between FTD syndromes^22-25^ and for ruling out non-FTLD cases.^26,27^ Structural magnetic resonance imaging (MRI) atrophy and [^18^F]fluorodeoxyglucose (FDG)-PET can identify patterns of atrophy and glucose hypometabolism, respectively, with both shared and complementary information on neurodegeneration in FTD syndromes.^22,28^  *In vivo* quantification of tau deposition poses challenges in FTD syndromes because of the absence of a radiotracer optimized for 3R and 4R tauopathies. Nevertheless, flortaucipir, which shows optimal binding to Alzheimer's disease 3R + 4R tau, also shows elevated uptake in FTD cortical and subcortical signature regions relative to the healthy population and shows differential patterns across FTD syndromes and consistency with 4R tau burden at autopsy.^29-33^

Unlike binary classification of neurodegenerative diseases versus controls, discriminating among FTD clinical syndromes utilizing information extracted from neuroimages has been proven challenging.^34^ Studies have used various statistical and machine learning methods, achieving only a sub-optimal discriminative accuracy with structural MRI^34,35^ and FDG-PET data.^36^ Image-based classifiers suffer from the risk of model overfitting since the number of variables (voxels) is orders of dimension higher than the number of participants. Hence, dimensionality reduction is imperative. Some of the most popular methods applied to neuroimages for dimensionality reduction are principal component analysis (PCA), relying on singular value decomposition,^36-38^ independent component analysis (ICA),^14^ and non-negative matrix factorization (NNMF).^39,40^ PCA and NNMF have revealed comparable capabilities for low-rank approximation of voxel-based grey matter segmentations of T1 structural MRI, although NNMF components had a better transferability to a new, unseen dataset.^40^ Each method captures different aspects of the same information: PCA components explain the most variance (i.e. orthogonal components), ICA components are statistically independent (e.g. different signals), while NNMF components are spatially colocalized features that covary across the dataset.^40^

We aimed to identify unsupervised data-driven patterns of multimodal imaging abnormalities in FTD syndromes. To address this research question, PCA was the appropriate data reduction technique. We evaluated how the principal components of each imaging modality captured syndrome-specific or shared patterns of abnormalities and how these patterns were differentially related to the clinical domains of impairment. Secondarily, we investigated the ability of classifiers based on imaging principal components to correctly assign clinical syndromes, with or without the aid of demographic and clinical information. We leveraged a large and diverse cohort of study participants who were clinically diagnosed with FTD syndromes and underwent a detailed clinical assessment and multimodal neuroimaging scans: structural MRI, FDG-PET, and [^18^F] flortaucipir-PET for tau deposition.

## Materials and methods

### Participants

Four hundred participants with a clinical FTD diagnosis were included in the study. All participants had been recruited by the Neurodegenerative Research Group (NRG) in the Department of Neurology, Mayo Clinic, between 2009 and 2024. The cohort consisted of participants meeting clinical criteria for bvFTD (*n* = 12),^3,4^ svPPA (*n* = 28),^4^ the right temporal variant of FTD (rtvFTD, *n* = 11),^41^ AOS-PAA (*n* = 70), PPAOS (*n* = 53),^5,6,8,42-44^ PSP (*n* = 185),^10^ and CBS (*n* = 23).^11^ Any patient who met criteria for PPA^8^ but did not fit into any other diagnostic category was classified as PPA-other (*n* = 18). This group consisted of amyloid-beta (Aβ)-negative participants with the logopenic variant of PPA, participants with agrammatic aphasia without apraxia of speech (PAA), and participants with PPA that could not be classified into one of the three well recognized variants. Participants with the semantic variant of PPA were independently categorized as svPPA, and participants with agrammatic aphasia who also had apraxia of speech were categorized as AOS-PAA. PPA participants (logopenic variant) were required to be Aβ-negative on a Pittsburgh Compound B PET scan to rule out Alzheimer’s disease.^45^ For the same reason, CBS participants were required to be either Aβ-PET negative or tau-PET negative. Participants diagnosed with any other FTD syndrome were included regardless of Aβ and tau positivity. Details on PSP clinical variants are reported in Supplementary Table 1. All 400 participants had a structural MRI, 279 had a tau-PET and 267 had an FDG-PET scan. Clinical diagnosis was obtained in a blinded manner to imaging results. Of the 400 participants, 127 had a neuropathological diagnosis. *APOE* genotype was generated as previously described.^46^

All participants underwent a detailed neurological and neuropsychological evaluation. Participants were enrolled in several different National Institutes of Health (NIH)-funded grants that had slightly different clinical batteries. Clinical tests that were collected relatively consistently across batteries included the Montreal Cognitive Assessment (MoCA)^47^ to assess general cognitive function; the MDS-sponsored revision of the Unified Parkinson's Disease Rating Scale (UPDRS) part III^48^ to assess motor parkinsonism; the Frontal Assessment Battery (FAB)^49^ to assess executive dysfunction; the 15-item Boston Naming Test (BNT)^50^ to assess confrontational naming; the Western Aphasia Battery (WAB)^51^ to quantify global language ability, including praxis, aphasia quotient (AQ), repetition, and animal fluency; the Letter Fluency Sum^52^ to assess phonemic fluency; the Apraxia of Speech rating Scale version 3 (ASRS-3)^53^ to quantify apraxia of speech; the PSP Rating Scale^54^ to quantify PSP disease severity; and the Pyramids and Palm Trees Test word-word version (PPT word-word) to evaluate single-word comprehension.^55^ Details of available clinical scores for each syndrome are reported in Supplementary Table 2.

One hundred and nine cognitively unimpaired participants (MoCA: 27 [26, 28]) were also recruited by the NRG between 2016 and 2024 and completed the same imaging protocols [structural MRI (*n* = 109), tau-PET (*n* = 95), FDG-PET (*n* = 44)]. The study was approved by the Mayo Clinic IRB, and all participants provided written informed consent to participate in this study.

### Image acquisition

All participants underwent a 3T head MRI protocol that included a magnetization-prepared rapid gradient echo (MPRAGE) sequence on either a GE or Siemens Prisma scanner (GE Healthcare, Milwaukee, Wisconsin or Siemens Healthcare, Erlangen, Germany).^56,57^ All PET scans were acquired using PET/CT on GE or Siemens scanners operating in 3D mode. For tau-PET, an intravenous bolus injection of approximately 370 MBq (range 333–407 MBq) of [^18^F] flortaucipir was administered, followed by a 20-min PET acquisition performed 80 min after injection. For FDG-PET, participants were injected with [^18^F] FDG of approximately 459 MBq (range 367–576 MBq) and, after a 30-min uptake period, an 8-min FDG-PET scan was acquired, consisting of four 2-min dynamic frames following a low-dose CT transmission scan. Standard corrections were applied. Emission data were reconstructed into a 256 × 256 matrix with a 30-cm field of view (in-plane pixel size = 1.0 mm). The Aβ-PET images to establish Aβ-positivity were acquired and processed as previously described.^58,59^

#### Structural MRI and PET preprocessing

Each MPRAGE was segmented into grey matter and bias-field corrected using Unified Segmentation^60^ in SPM12 (Wellcome Trust Centre for Neuroimaging, London, UK), with Mayo Clinic Adult Lifespan Template (MCALT) (https://www.nitrc.org/projects/mcalt/) tissue priors and settings.^61^ PET images were rigidly registered to the corresponding MPRAGE using ANTs. PET standard uptake value ratios (SUVR) were then calculated in voxels segmented as grey or white matter with the cerebellar crus (flortaucipir-PET) or the pons (FDG-PET) grey matter as the reference region. The grey and white matter segmentation maps for each participant were summed into one tissue volume map. PET and MR images of each subject were subsequently spatially normalized to the MCALT template and smoothed with a 3- and 8-mm full-width at half-maximum kernel, respectively, for the voxel-based analyses.

### Statistical analysis

Participant characteristics were compared to cognitively unimpaired participants and between syndromes with Fisher’s exact test or chi-square test for categorial variables and Wilcoxon rank sum test or Kruskal-Wallis test for continuous variables.

#### Voxel-based morphometry

SPM12 was used to perform multiple regression analyses that assessed differences in MRI grey and white matter segmentation, FDG-PET uptake, and tau-PET uptake relative to the cognitively unimpaired participants, while adjusting for age and sex effects. Scanner manufacturer was included as a covariate in the MRI analyses. Results were extracted at *P* < 0.05 with FWE correction for multiple comparisons.

#### Principal component analyses

PCA was performed separately on MRI, FDG-PET, and tau-PET images with the PCA function in MATLAB version R2022a. Given *n* images, PCA yields *n-1* principal components. To disregard the influence of normal ageing, sex, total intracranial volume (TIV), and scanner manufacturer (on MRI only), the images were *w*-scored before applying PCA, using SPM. W-scoring consists of deriving a linear relationship between the images of the cognitively unimpaired participants and the covariates of interest (age, sex, scanner manufacturer, and TIV). The linear regression coefficients are then applied to the FTD participants’ characteristics (age, sex, TIV, and scanner manufacturer) to obtain a predicted image representing the ‘healthy’ version of the FTD participants. The *w*-scored images were obtained as the difference between the real image and the predicted image, divided by the standard deviation of the residuals from the linear regression.^62^ Voxel-based representations of the first four principal components for each modality were constructed using the coefficients from the PCA function. Participant-specific principal components scores were plotted by syndrome and compared across all syndromes using ANOVA and between each pair of syndromes using *t*-tests. Linear regression on principal components and clinical scores, covarying for age and sex, was run to determine the association between the two.

#### Predictive analyses

The glmnet package in R was employed to fit penalized multinomial logistic regression models with syndrome as the outcome. Eight different models were evaluated, using as predictors: (i) all MRI-based principal components; (ii) all tau-PET-based principal components; (iii) all FDG-PET-based principal components; (iv) MRI-based principal components plus demographics and clinical measures (age, sex, MoCA, MDS-UPDRS III, and FAB scores); (v) tau-PET-based principal components plus demographics and clinical measures; (vi) FDG-PET-based principal components plus demographics and clinical measures; (vii) MRI, tau-PET and FDG-PET-based principal components (*n* = 12 for each modality); (viii) MRI, tau-PET and FDG-PET-based principal components (*n* = 12 for each modality) plus demographics and clinical measures. Among the available clinical tests, only MoCA, MDS-UPDRS III, and FAB scores were included in the models because these scores were available for over 80% of participants for each syndrome. A control model with only demographics and clinical measures as predictors was also run to facilitate the interpretation of the performance metrics obtained with the imaging-based principal components as predictors.

When principal components from different imaging modalities were combined, the first 12 were included because they explained at least 1% of the variability in the images. Alpha, the elastic net penalty in glmnet, balances L1 (lasso) and L2 (ridge) regularization, and it was set to 0.9 (L1 > L2) in the models including all principal components as predictors (i.e. dropping most predictors) and 0.5 (L1∼L2) in the models including 12 principal components. The lambda parameter, which determines regularization strength, was optimized using a leave-one-out cross-validation for each model. The performance of the multiclass classifiers was evaluated with the area under the receiver operating characteristic curve (AUROC), using the R library pROC (multiclass.roc function). Sensitivity (i.e. true positive rate) and specificity (i.e, true negative rate) for each syndrome were also calculated. The 5–95% confidence intervals (CIs) on the AUROC were obtained using a stratified bootstrap with 5000 replicates.

To test the predictive capabilities of the models, a cross-validation scheme was implemented. Namely, the full sample of participants was randomly split into a training set and a testing set, with an 80:20 proportion, repeating the process 100 times. The 80:20 proportion was forced on each syndrome, given our imbalanced dataset. Each time, the model was fitted in the training set and then used to predict the syndromes in the testing set. The predictive capabilities of the models were evaluated on the test sets as mean sensitivity and mean specificity for each syndrome and mean AUROC across the 100 runs.

Given the strong imbalance of our cohort towards PSP, we also ran the imaging-based logistic regression models with two alternative strategies. First, we ran the models on a down-sampled cohort that included 70 PSP for MRI, 55 for FDG-PET, and 40 for tau-PET, matching the group size of the second-most frequent syndrome, which was AOS-PAA. The PSP group was down-sampled using random selection. All participants from all other syndromes were included. These models were run, including the first 100 PCs as predictors. Moreover, in an exploratory way, we ran a weighted logistic regression on MRI-based principal components, assigning to each clinical syndrome a weight that was the reciprocal of the group size relative to the full cohort.

These analyses were run in R version 4.2.2.

## Results

### Participants

Demographics and clinical characteristics of the participants are reported in Table 1. Age and disease duration differed significantly across syndromes, with PPAOS participants being the oldest, rtvFTD the youngest with the longest disease duration, and PPA-other participants having the shortest disease duration. No difference was present in sex, education, and *APOE* e4 status. Clinical scores differed significantly across the syndromes, which showed the expected clinical impairment. Poor performance was noted on the MoCA and FAB in bvFTD, on MDS-UPDRS III, PSP Rating Scale, and WAB praxis in PSP, CBS, and AOS-PAA, on ASRS-3 in AOS-PAA, PPAOS, and CBS, on the BNT and WAB animal fluency in svPPA, and on the PPT word-word in svPPA and rtvFTD participants. Pathological diagnoses included PSP, corticobasal degeneration (CBD), TDP-43, MSA (multiple system atrophy), mitochondrial encephalopathy with lactic-acidosis and stroke-like episodes syndrome (MELAS), Lewy Body disease (LBD), Pick’s disease (PiD), globular glial tauopathy (GGT), diffuse neurofibrillary tangles with calcification (DNTC), Alzheimer’s disease neuropathological changes (ADNCs), and mixed 3R/4R tauopathy. (Supplementary Table 2). The number and percentage of participants for each diagnosis who underwent each clinical test are reported in Supplementary Table 3.

**Table 1:** Demographic and clinical characteristics

	bvFTD	svPPA	rtvFTD	AOS-PAA	PPAOS	PPA-other	PSP	CBS	Overall P value
Demographics									
* n*	12	28	11	70	53	18	185	23	-
* Age (y*ea*rs)*	66, (61.5, 72.7)	68.2, (59.7, 71.6)	64.2, (60.9, 69.3)	69, (62.9, 73.4)	72.7, (63.1, 77.3)	65.8, (62.8, 72.5)	70.4, (65.9, 75.4)	65.4, (59.8, 68.5)	0.003
* Disease duration (y*ea*rs)*	3.6, (2.3, 4)	4.3, (3, 5.8)	4.7, (4.4, 5.1)	3.8, (2.6, 5.6)	3.3, (2.1, 5.2)	2.1, (2, 3.1)	3.7, (2.5, 5.2)	2.8, (1.7, 3.4)	0.009
* Male (n, %)*	7, 58%	12, 43%	5, 45%	34, 49%	25, 47%	6, 33%	97, 52%	12, 52%	0.822
* Education (y*ea*rs)*	16, (14, 16)	16, (13, 16)	16, (15, 18)	15, (12, 16)	16, (14, 18)	15, (12, 16)	14, (12, 16)	15, (12, 16)	0.058
* APOE E4 carriers (n, %)*	1, 14%	10, 38%	4, 44%	20, 31%	6, 15%	4, 22%	28, 25%	5, 71%	0.035
* Aβ positive/negative*	2/9	9/13	4/5	12/45	14/33	3/12^b^	36/80	1/20	0.079
Imaging									
* FDG-PET*	9	23	4	55	45	15	106	10	-
* Tau-PET*	12	18	8	40	30	8	140	23	-
Clinical tests									
* MoCA*	16 (14.5, 22)	19 (14, 21.5)	22.5 (18.2, 24)	21 (17, 25)	27 (25, 28)	22 (18, 24)	23 (19, 26)	23 (21, 24)	*P* < 0.001
* MDS-UPDRS III*	10 (2, 30.5)	0 (0, 2)	2 (0, 3.5)	17 (10, 25)	12 (5, 22)	3.5 (1, 7.2)	41 (30.5, 54.5)	29 (22.8, 40.5)	*P* < 0.001
* PSP rating scale*	7 (4.5, 18.5)	5 (3, 6.5)	2 (1, 3)	18 (10, 28.5)	8 (4, 17)	6 (2.5, 10.2)^a^	37 (28.5, 46)	24 (20, 27.5)	*P* < 0.001
* FAB*	13 (8, 14.2)	15 (12.8, 16.2)	16 (15.5, 18)	13 (10, 16)	17 (15, 17)	14 (10, 16)	14 (11, 16)	15 (13, 16.5)	*P* < 0.001
* ASRS total v3*	5 (2, 6)	0 (0, 1)	0 (0, 0)	22 (12, 29)	16 (11.5, 23)	1 (0, 3)	4 (2, 6.8)	20 (15, 26)	*P* < 0.001
* BNT*	12 (8, 12)^a^	0 (0, 2)^a^	10.5 (10.2, 10.8)^a^	12 (10.5, 14)	14 (13, 15)	10 (6.5, 13)	14 (12, 14)	13 (11.8, 14)	*P* < 0.001
* Letter fluency sum*	11 (10, 11)	17.5 (12.2, 27.2)	21 (19, 30)	10 (6, 19)	20 (12.8, 31.2)	13 (6.5, 23.5)	5.5 (3.8, 7)^a^	9 (3.5, 17)^a^	*P* < 0.001
* WAB praxis*	59 (47, 60)	57.5 (48, 59.2)	60 (59, 60)	52 (45, 57)	58 (55, 59.2)	56 (53.8, 58.2)	50 (37, 52)^a^	51 (49, 55)	*P* < 0.001
* WAB AQ*	90.3 (87.2, 93.3)	80.4 (64.4, 91.7)	91.2 (87.6, 94.2)	85.2 (77.5, 92.6)	97.8 (96.6, 99.2)	89.3 (79.3, 92.7)	97 (93.6, 98.1)^a^	94.6 (92.5, 96.5)	*P* < 0.001
* WAB repetition*	9.2 (9, 9.8)	8.9 (7.8, 9.3)	9.6 (9.2, 9.7)	8.8 (7.8, 9.5)	9.7 (9.4, 9.9)	9.1 (8.2, 9.6)	9.6 (9.4, 10)^a^	9 (8.6, 9.5)^a^	*P* < 0.001
* WAB animal fluency*	10 (9, 12)	7 (3, 9)	8 (6.5, 10)	11 (7, 14)	16 (14.2, 20)	9 (7, 12)	12 (7.5, 14)^a^	14.5 (11.2, 15)	*P* < 0.001
PPT word-word	47.5 (46.2, 48.8)	39 (32, 43.5)	38.5 (34.2, 41.5)	49 (48, 50)	50 (49.2, 51)	48 (46, 51)	49 (49, 49)^a^	47 (47, 47)^a^	*P* < 0.001

Demographic and clinical data are reported as median (25th, 75th quartiles), number, and percentage. *P*-values are from Kruskal-Wallis and Fisher’s exact tests. AOS-PAA, apraxia of speech—primary agrammatic aphasia; APOE E4, Apolipoprotein E4; ASRS, Apraxia of speech rating scale; BNT, Boston naming test; bvFTD, behavioural variant of frontotemporal dementia; CBS, corticobasal syndrome; FAB, frontal assessment battery; FDG, fluorodeoxyglucose; GM, grey matter; MDS-UPDRS, Movement Disorder Society-sponsored revision of the Unified Parkinson’s Disease Rating Scale; MoCA, Montreal Cognitive Assessment; PPA, primary progressive aphasia; PPAOS, primary progressive apraxia of speech; PPT, pyramid and palm trees test; PSP, progressive supranuclear palsy; rtvFTD, right temporal variant of frontotemporal dementia; svPPA, semantic variant of primary progressive aphasia; WAB AQ, Western aphasia battery aphasia quotient; WM, white matter. ^a^Score available for less than 50% of participants. ^b^All three A*β*-positive PPA-other were classified as PAA.

### Voxel-level analyses

**Figure 1:**
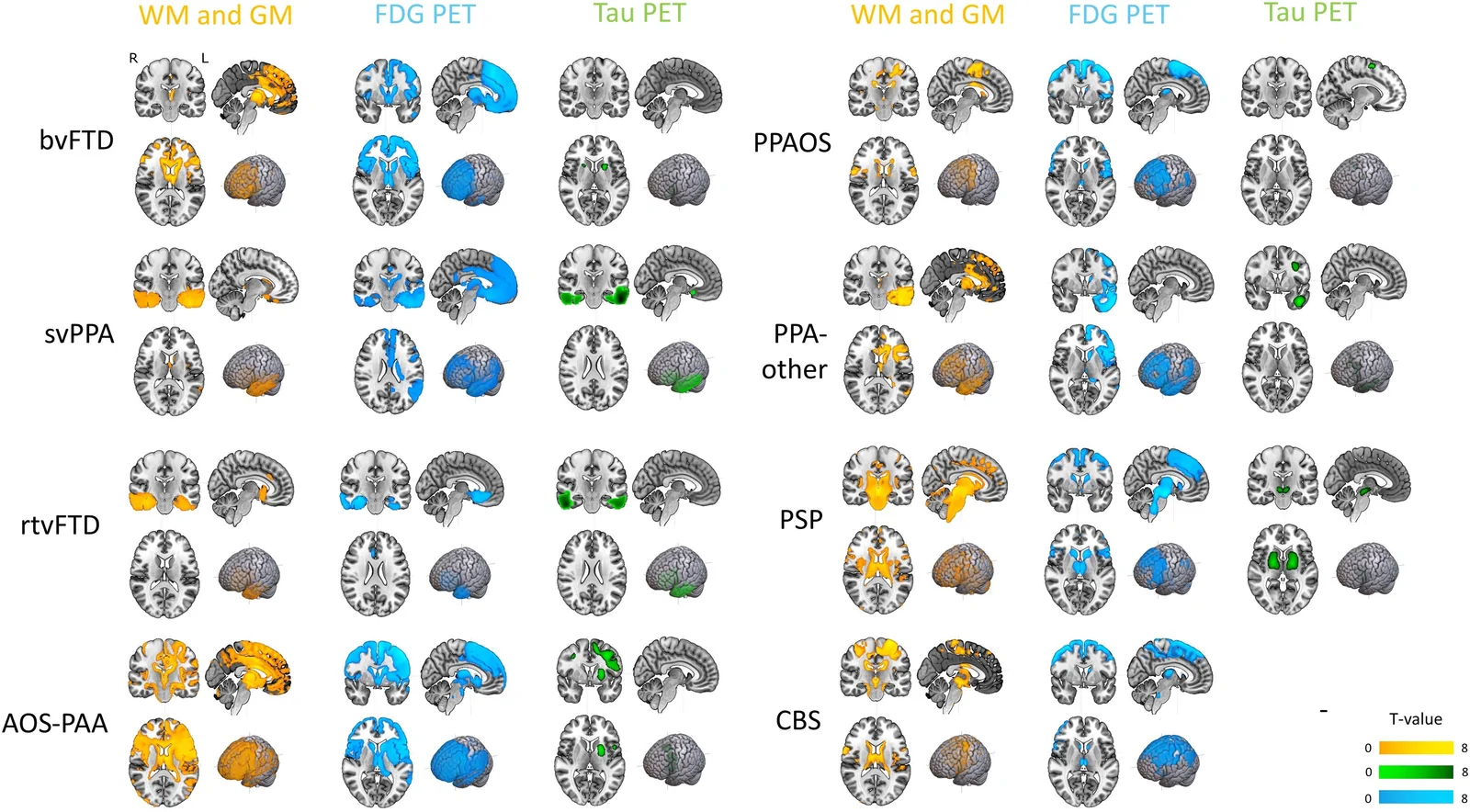
**Patterns of atrophy, hypometabolism, and tau-PET uptake for each syndrome.** Voxel-based maps of grey and white matter atrophy (orange), lower FDG-PET uptake (blue), and higher tau-PET uptake (green) in each syndrome were obtained from multiple regressions relative to cognitively unimpaired individuals (structural MRI *n* = 109, tau-PET *n* = 95, FDG-PET *n* = 44). Please refer to Table 1 for the size cohort of each syndrome in each imaging modality. Results are reported at *P* < 0.05, FWE, covarying for age, sex, and, for MRI analyses, scanner manufacturer. AOS-PAA, apraxia of speech—primary agrammatic aphasia; bvFTD, behavioural variant of frontotemporal dementia; CBS, corticobasal syndrome; GM, grey matter; PPA, primary progressive aphasia; PSP, progressive supranuclear palsy; PPAOS, primary progressive apraxia of speech; rtvFTD, right temporal variant of frontotemporal dementia; svPPA, semantic variant of primary progressive aphasia; WM, white matter.

Compared to cognitively unimpaired participants, bvFTD participants showed grey and white matter atrophy and hypometabolism in the basal ganglia and frontal lobe, including the supplementary motor area, and elevated tau-PET uptake only in the basal ganglia (Fig. 1). svPPA participants showed atrophy in the bilateral temporal lobe, with some inferior frontal involvement, hypometabolism in the frontotemporal lobes, and elevated tau-PET uptake in the temporal cortex, with stronger involvement of the left hemisphere (Fig. 1). rtvFTD participants showed atrophy and hypometabolism in the temporal lobe, predominantly on the right, and in the inferior frontal gyrus, with elevated tau-PET uptake in the bilateral temporal lobes (Fig. 1). AOS-PAA participants showed atrophy in the cerebellum, midbrain, basal ganglia, temporal and frontoparietal cortices, and a similar pattern of hypometabolism, with extensive abnormalities in the supplementary motor area (Fig. 1). Elevated tau-PET uptake in AOS-PAA was present in the basal ganglia, precentral gyrus, supplementary motor area, and middle and inferior frontal gyri (Fig. 1). PPAOS participants had atrophy and hypometabolism in the basal ganglia and frontal lobe, including the supplementary motor area, and elevated tau-PET uptake limited to the supplementary motor area (Fig. 1). PPA-other participants showed atrophy and hypometabolism in the left temporal and frontoparietal cortex and basal ganglia, and elevated tau-PET uptake in the left temporal lobe and the left middle frontal gyrus (Fig. 1). PSP participants showed atrophy and hypometabolism in the cerebellum, midbrain, basal ganglia, and frontal lobe, including the supplementary motor area, and elevated tau-PET uptake in the basal ganglia, midbrain, and dentate nucleus of the cerebellum (Fig. 1). CBS participants showed atrophy and hypometabolism in the midbrain, basal ganglia, and frontoparietal cortex, including the supplementary motor area (Fig. 1). Elevated tau-PET uptake in CBS participants was noted in the superior frontal gyrus, paracentral lobule, and temporal lobes, but it did not survive the established threshold of *P* < 0.05 FWE.

### Principal component analysis

#### MRI-based principal components

**Figure 2:**
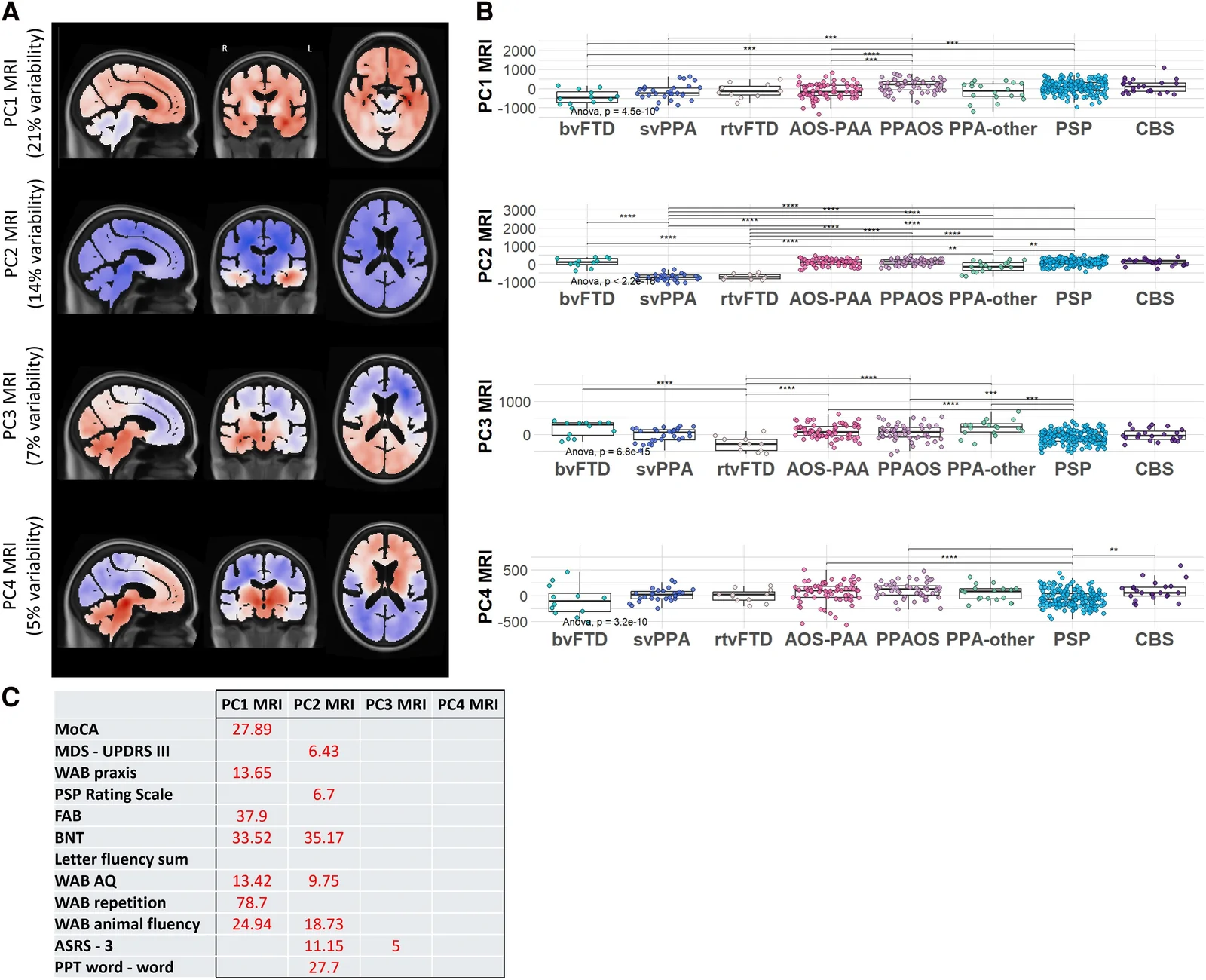
**MRI principal components.** First four principal components of grey and white matter MRI *w*-scored segmentation images (**A**). Principal component images are constructed using the coefficients (i.e. loadings) from principal component analysis. Participant–specific principal components scores are plotted grouped by syndrome and compared across all syndromes using ANOVA and between each pair of syndromes using t-tests. Each individual datapoint represents the principal component score of one participant. Only significant comparisons at *P* < 0.05 after Bonferroni correction are shown (**P < 0.01. ***P < 0.001. ****P < 0.0001) (**B**). Beta estimates from linear regression on principal components and clinical scores, covarying for age and sex. Only coefficients with *P* < 0.05 after Bonferroni correction for multiple comparisons are reported (**C**). Please refer to Table 1 for the size cohort of each syndrome. AOS-PAA, apraxia of speech—primary agrammatic aphasia; ASRS, Apraxia of speech rating scale; BNT, Boston naming test; bvFTD, behavioural variant of frontotemporal dementia; CBS, corticobasal syndrome; FAB, frontal assessment battery; GM, grey matter; MDS-UPDRS, Movement Disorder Society-sponsored revision of the Unified Parkinson’s Disease Rating Scale; MoCA, Montreal Cognitive Assessment; PPA, primary progressive aphasia; PPAOS, primary progressive apraxia of speech; PPT, pyramid and palm trees test; PSP, progressive supranuclear palsy; rtvFTD, right temporal variant of frontotemporal dementia; svPPA, semantic variant of primary progressive aphasia; WAB AQ, Western aphasia battery aphasia quotient; WM, white matter.

The first MRI principal component (PC1 MRI) explained 21% of the variability in the images (Fig. 2A, top row) and captured widespread cortical atrophy (red areas) with the highest coefficients in the frontal lobe, particularly in the insular, orbitofrontal, and cingulate cortices, and in the temporal lobe, particularly in the middle temporal and anteromedial temporal cortex. PC1 MRI also captured subcortical atrophy in the basal ganglia, mainly in the putamen. bvFTD had the lowest median scores, while PPAOS had the highest (Fig. 2B, top row), but participants of each syndrome showed large variability in their scores. Lower PC1 MRI scores (i.e. more atrophy) were associated with worse performance on the MoCA, FAB, language tests (BNT, WAB-AQ, WAB repetition, WAB animal fluency), and WAB praxis (Fig. 2C).

The second MRI principal component (PC2 MRI) explained 14% of the variability and captured atrophy in the temporal lobe (red), particularly in the superior and middle temporal poles, inferior temporal, amygdala, entorhinal cortex, fusiform, and hippocampus, with higher coefficients on the left side, versus atrophy in the precentral gyrus, supplementary motor area, midbrain, subthalamic nucleus, red nucleus, and dentate nucleus of the cerebellum (blue) (Fig. 2A, second row). rtvFTD and svPPA had the lowest median scores, followed by PPA-other, while AOS-PAA, PPAOS, PSP, and CBS had higher median scores (Fig. 2B, second row). Lower PC2 MRI scores (i.e. more temporal atrophy) were associated with worse performance on speech-language (BNT, WAB-AQ, and WAB animal fluency) and semantic (PPT word-word) tests, while higher scores (i.e. more atrophy in the blue areas) were associated with worse performance on motor tests (MDS-UPDRS-III and PSP Rating Scale) and worse apraxia of speech (ASRS-3) (Fig. 2C).

The third MRI principal component (PC3 MRI) explained 7% of the variability and captured atrophy in the cerebellum, midbrain, and right temporal lobe (red) versus atrophy in the left frontal lobe (blue) (Fig. 2A, third row). PSP and rtvFTD had lower median scores, while PPAOS, PPA-other, AOS-PAA, and bvFTD had higher median scores (Fig. 2B, third row). Higher scores were associated with worse apraxia of speech (ASRS-3) (Fig. 2C).

The fourth MRI principal component (PC4 MRI) explained 5% of the variability and captured atrophy in the midbrain, including substantia nigra and red nucleus, thalamus, cerebellum, basal ganglia, and frontal cortex (red) versus atrophy in the parietal lobe, particularly the postcentral gyrus (blue) (Fig. 2A, fourth row). PSP had statistically lower median scores than AOS-PAA, PPAOS, and CBS (Fig. 2B, fourth row). PC4 MRI scores were not associated with clinical scores (Fig. 2C).

#### FDG-PET-based principal components

**Figure 3:**
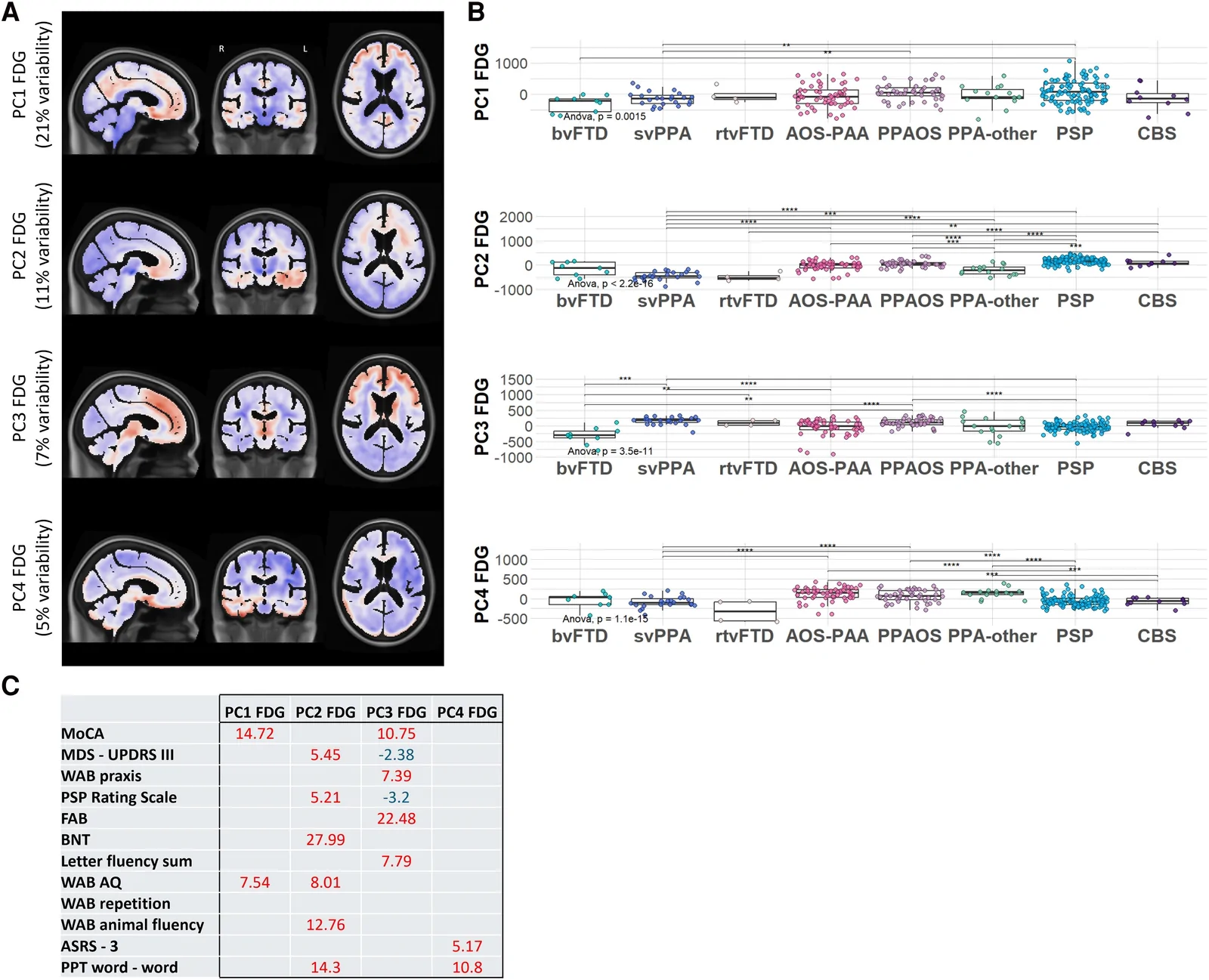
**FDG-PET principal components.** First four principal components of *w*-scored SUVR images of FDG-PET (**A**). Principal component images are constructed using the coefficients (i.e. loadings) from principal component analysis. Participant–specific principal components scores are plotted grouped by syndrome and compared across all syndromes using ANOVA and between each pair of syndromes using t-tests. Each individual datapoint represents the principal component score of one participant. Only significant comparisons at *P* < 0.05 after Bonferroni correction are shown (**P < 0.01. ***P < 0.001. ****P < 0.0001) (**B**). Beta estimates from linear regression on principal components and clinical scores, covarying for age and sex. Only coefficients with *P* < 0.05 after Bonferroni correction for multiple comparisons are reported (**C**). Please refer to Table 1 for the size cohort of each syndrome. AOS-PAA, apraxia of speech—primary agrammatic aphasia; ASRS, Apraxia of speech rating scale; BNT, Boston naming test; bvFTD, behavioural variant of frontotemporal dementia; CBS, corticobasal syndrome; FAB, frontal assessment battery; GM, grey matter; MDS-UPDRS, Movement Disorder Society-sponsored revision of the Unified Parkinson’s Disease Rating Scale; MoCA, Montreal Cognitive Assessment; PPA, primary progressive aphasia; PPAOS, primary progressive apraxia of speech; PPT, pyramid and palm trees test; PSP, progressive supranuclear palsy; rtvFTD, right temporal variant of frontotemporal dementia; svPPA, semantic variant of primary progressive aphasia; WAB AQ, Western aphasia battery aphasia quotient; WM, white matter.

The first FDG-PET principal component (PC1 FDG) explained 21% of the variability and captured hypometabolism in the inferior frontal, particularly the orbital gyrus, insular, parietal (precuneus), and temporal cortices (red) versus hypometabolism in the midbrain (blue) (Fig. 3A, top row). bvFTD and svPPA showed lower median scores, while PSP and PPAOS showed higher median scores (Fig. 3B, top row). Lower PC1 scores (i.e. lower metabolism in red areas) were associated with worse performance on the MoCA and WAB AQ (Fig. 3C).

The second FDG-PET principal component (PC2 FDG) explained 11% of the variability and captured hypometabolism in the left temporal and left orbitofrontal cortex (red) versus hypometabolism in the midbrain, thalamus, and occipital cortex (blue) (Fig. 3A, second row). rtvFTD and svPPA showed lower median scores, while CBS, PSP, AOS-PAA, and PPAOS showed higher scores (Fig. 3B, second row). Higher scores were associated with worse performances on motor tests (MDS-UPDRS III, PSP Rating Scale), while lower scores were associated with worse performance on language (BNT, WAB-AQ, and WAB animal fluency), including semantics (PPT word-word) (Fig. 3C).

The third FDG-PET principal component (PC3 FDG) explained 7% of the variability and captured hypometabolism in the superior and middle frontal lobe, including the supplementary motor area, operculum, basal ganglia (caudate), midbrain, and thalamus (red) (Fig. 3A, third row). bvFTD had the lowest median scores, while PPAOS, rtvFTD, and svPPA showed higher median scores (Fig. 3B, third row). Lower scores were associated with worse performance on MoCA, FAB, letter fluency, and WAB praxis, while higher scores were associated with worse performance on motor tests (MDS-UPDRS III and PSP Rating Scale) (Fig. 3C).

The fourth FDG-PET principal component (PC4 FDG) explained 5% of the variability and captured hypometabolism in the right temporal lobe (red) versus hypometabolism in the precentral and supplementary motor area regions (blue) (Fig. 3A, fourth row). rtvFTD showed the lowest median score, while AOS-PAA, PPA-other, and PAOS had the highest (Fig. 3B, fourth row). Higher scores were associated with worse performance on the apraxia of speech test (ASRS-3), while lower scores were associated with worse performance on the semantic test (PPT word-word) (Fig. 3C).

#### Tau-PET-based principal components

**Figure 4:**
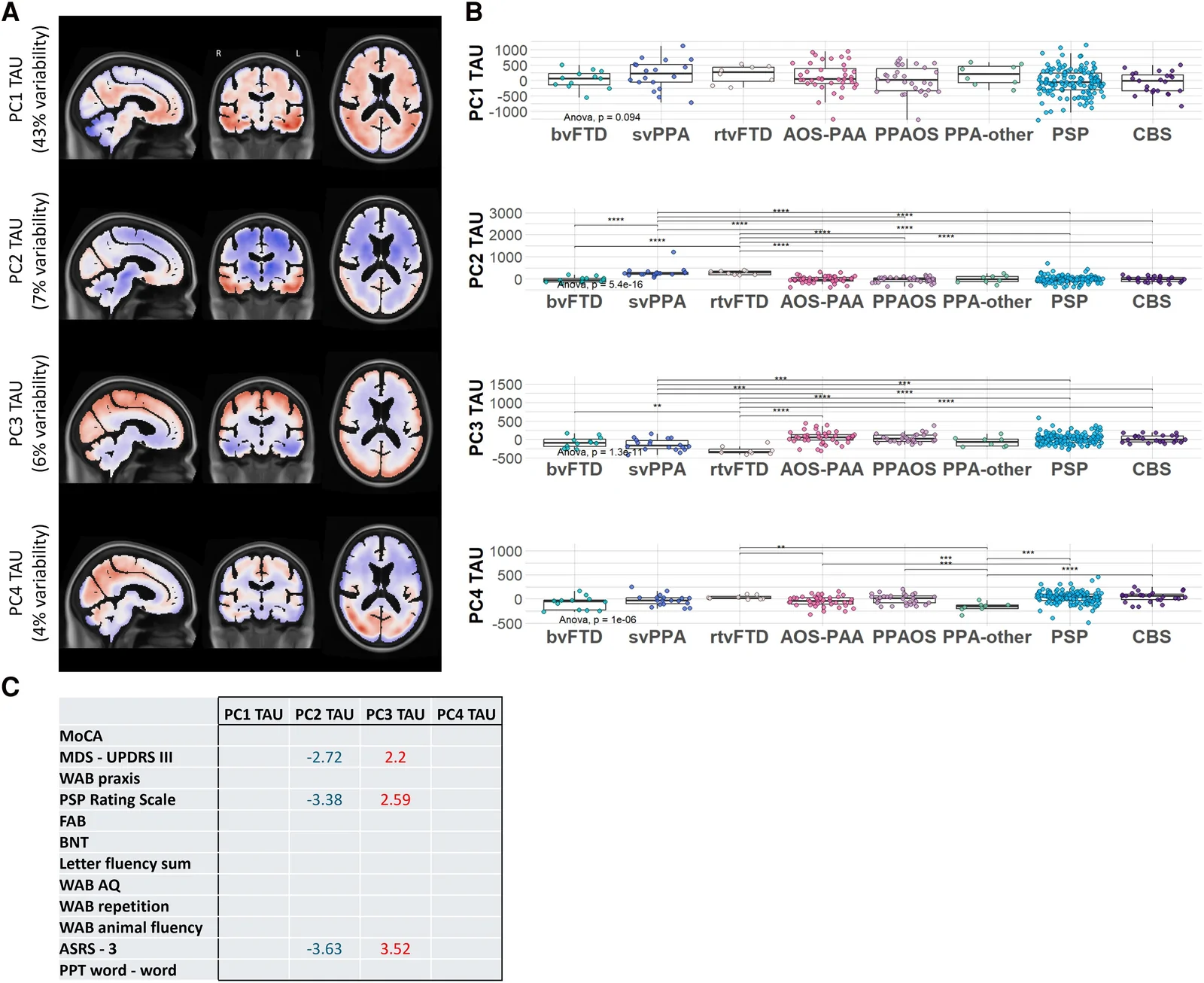
**Tau-PET principal components.** First four principal components of *w*-scored SUVR images of tau-PET (**A**). Principal component images are constructed using the coefficients (i.e. loadings) from principal component analysis. Participant-specific principal components scores are plotted grouped by syndrome and compared across all syndromes using ANOVA and between each pair of syndromes using t-tests. Each individual datapoint represents the principal component score of one participant. Only significant comparisons at *P* < 0.05 after Bonferroni correction are shown (**P < 0.01. ***P < 0.001. ****P < 0.0001) (**B**). Beta estimates from linear regression on principal components and clinical scores, covarying for age and sex. Only coefficients with *P* < 0.05 after Bonferroni correction for multiple comparisons are reported (**C**). Please refer to Table 1 for the size cohort of each syndrome. AOS-PAA, apraxia of speech—primary agrammatic aphasia; ASRS, Apraxia of speech rating scale; BNT, Boston naming test; bvFTD, behavioural variant of frontotemporal dementia; CBS, corticobasal syndrome; FAB, frontal assessment battery; GM, grey matter; MDS-UPDRS, Movement Disorder Society-sponsored revision of the Unified Parkinson’s Disease Rating Scale; MoCA, Montreal Cognitive Assessment; PPA, primary progressive aphasia; PPAOS, primary progressive apraxia of speech; PPT, pyramid and palm trees test; PSP, progressive supranuclear palsy; rtvFTD, right temporal variant of frontotemporal dementia; svPPA, semantic variant of primary progressive aphasia; WAB AQ, Western aphasia battery aphasia quotient; WM, white matter.

The first tau-PET principal component (PC1 TAU) explained 43% of the variability and represented a widespread presence of tau (as measured by flortaucipir uptake), with the highest intensity in the bilateral temporal lobes and frontal cortex (red) (Fig. 4A, top row). Median scores did not differ across the syndromes (Fig. 4B, top row) and were not associated with clinical tests (Fig. 4C).

The second tau-PET principal component (PC2 TAU) explained 7% of the variability and captured elevated tau-PET uptake in the lateral and medial temporal lobe bilaterally (red) versus uptake in the midbrain, thalamus, basal ganglia, precentral gyrus, and the supplementary motor area (blue) (Fig. 4A, second row). rtvFTD and svPPA had the highest median scores (Fig. 4B, second row). Lower scores were associated with worse performance on the apraxia of speech (ASRS-3) and motor (MDS-UPDRS III and PSP Rating Scale) tests (Fig. 4C).

The third tau-PET principal component (PC3 TAU) explained 6% of the variability and captured elevated tau-PET uptake in the parieto-occipital lobes, particularly in the precentral gyrus (red), versus uptake in the temporal lobe (blue) (Fig. 4A, third row). rtvFTD had the lowest median score, while AOS-PPA and PPAOS had the highest (Fig. 4B, third row). Higher scores were associated with worse performance on motor (MDS-UPDRS III and PSP Rating Scale) and apraxia of speech (ASRS-3) tests (Fig. 4C).

The fourth tau-PET principal component (PC4 TAU) explained 4% of the variability and captured elevated tau-PET uptake predominantly in the occipital lobe, but also in the cerebellum, right temporal and paracentral regions, supplementary motor area, and precuneus (red) (Fig. 4A, fourth row). PPA-other had the lowest median score, while PSP, PPAOS, and CBS had the highest (Fig. 4B, fourth row). PC4 scores were not associated with clinical tests (Fig. 4C).

### Performance of the imaging-based GLM models

All models based on imaging principal components alone showed an AUROC of at least 0.80, with MRI-based components reporting an AUROC of 0.86, FDG-PET reporting an AUROC of 0.80 and tau-PET reporting an AUROC of 0.88 (Table 2). The model based on only demographics (age, sex) and clinical scores (MoCA, MDS-UPDRS III, FAB) showed an AUROC of 0.64 (CI: 0.57, 0.69). On cross-validation, the AUROC values in the testing sets, obtained by averaging over 100 runs, were lower but comparable (Table 2). The sensitivity and specificity for each specific FTD syndrome are shown in Table 3, and Supplementary Figs 1–3 show how the syndromes were assigned to the participants by the classifiers.

**Table 2:** Classifiers performance on the full cohort

Classifier type	AUROC (model)	AUROC (cross-validation)
MRI (all PCs), *n* = 400	0.86 (CI: 0.83, 0.89)	0.76
MRI (all PCs) and Moca, UPDRS III, FAB, age, sex*, n* = 377	0.90 (CI: 0.87, 0.94)	0.78
FDG-PET (all PCs), *n* = 267	0.80 (CI: 0.71, 0.85)	0.79
FDG-PET (all PCs) and Moca, UPDRS III, FAB, age, sex, *n* = 252	0.89 (CI: 0.83, 0.93)	0.80
Tau-PET (all PCs), *n* = 279	0.88 (CI: 0.82, 0.91)	0.71
Tau-PET (all PCs) and Moca, UPDRS3, FAB, age, sex, *n* = 259	0.88 (CI: 0.81, 0.91)	0.76
FDG-PET, Tau-PET, MRI (12 PCs), *n* = 194	0.83 (CI: 0.76, 0.87)	0.78
FDG-PET, Tau-PET, MRI (12 PCs), and MoCa, UPDRS III, FAB, age, sex, *n* = 180	0.87 (CI: 0.81, 0.92)	0.80

The performance of each multiclass classifier is reported as AUROC (area under the receiver operating characteristic curve). In the left column, the AUROC is reported for the model along with the 5–95% confidence intervals (CIs) obtained with a stratified bootstrap with 5000 replicates. In the right column, the AUROC is obtained with cross-validation, to test the predictive capabilities of the models, averaging across the testing samples over 100 runs of cross-validation on randomized training and testing splits (80:20). Classifiers were built including: all MRI-based principal components; all FDG-PET-based principal components; all tau-PET-based principal components; all imaging-based principal components, demographics (age, sex), and clinical scores (MoCA, MDS-UPDRS III, FAB); first 12 principal components (explaining more than 1% variability each) from MRI, or FDG-PET, or tau-PET; first 12 principal components from MRI, FDG-PET, and tau-PET with or without demographics and clinical scores. The number of participants is reported next to each classifier type. FAB, frontal assessment battery; FDG, fluorodeoxyglucose; MDS-UPDRS, Movement Disorder Society-sponsored revision of the Unified Parkinson’s Disease Rating Scale; MoCA, Montreal Cognitive Assessment.

**Table 3:** Sensitivity and specificity of the classifiers for each syndrome on the full cohort

	Model		Cross-validation	
	Sensitivity	Specificity	Sensitivity	Specificity
**MRI PCs**				
bvFTD	25%	100%	1%	100%
svPPA	92.90%	99.50%	80%	97.60%
rtvFTD	90.90%	100%	51.70%	99.60%
AOS-PAA	87.10%	97.30%	44.50%	89.60%
PPAOS	69.80%	98.80%	20.10%	95%
PPA-other	38.90%	100%	2%	99.70%
PSP	99.50%	77.20%	93.60%	56.20%
CBS	34.80%	100%	0.40%	100%
**FDG-PET PCs**				
bvFTD	55.60%	100%	16%	100%
svPPA	100%	98%	83.20%	96.80%
rtvFTD	25%	100%	1%	100%
AOS-PAA	87.30%	93.90%	54.10%	85.30%
PPAOS	82.20%	96.40%	29.80%	90%
PPA-other	26.70%	100%	8%	99.30%
PSP	98.10%	88.20%	92.60%	75%
CBS	0%	100%	0%	100%
**Tau-PET PCs**				
bvFTD	58.30%	100%	3.30%	99.40%
svPPA	88.90%	99.20%	60.50%	97.20%
rtvFTD	87.50%	100%	21.50%	99.60%
AOS-PAA	92.50%	99.20%	28.20%	91.80%
PPAOS	70%	99.20%	2.80%	96.20%
PPA-other	50%	100%	0%	99.90%
PSP	100%	82%	96.50%	44.10%
CBS	69.60%	100%	3.20%	99.20%
**MRI, FDG-PET, Tau-PET (first 12 PCs)**				
bvFTD	55.60%	99.50%	29%	98.90%
*svPPA*	92.30%	98.90%	91.70%	98.80%
rtvFTD	66.70%	100%	-	-
AOS-PAA	66.70%	96.40%	47.50%	91%
PPAOS	63.60%	96.50%	27%	94.90%
PPA*-*other	42.90%	100%	9%	99.60%
PSP	99%	78%	97.40%	66.80%
CBS	30%	100%	0.50%	99.50%

Sensitivity and specificity of the classifiers were obtained for each syndrome (left) and the predictive capabilities of the classifiers were evaluated with cross-validation (right). For cross-validation, sensitivity and specificity for each diagnosis were calculated as the average value across the testing samples over 100 runs on randomized training and testing splits. Classifiers were built, including all MRI-based principal components (A); all FDG-PET-based principal components (B); all tau-PET-based principal components (C); first 12 principal components from MRI, FDG-PET, and tau-PET (D). AOS-PAA, apraxia of speech—primary agrammatic aphasia; APOE E4, Apolipoprotein E4; ASRS, Apraxia of speech rating scale; BNT, Boston naming test; bvFTD, behavioural variant of frontotemporal dementia; CBS, corticobasal syndrome; FAB, frontal assessment battery; FDG, fluorodeoxyglucose; GM, grey matter; MDS-UPDRS, Movement Disorder Society-sponsored revision of the Unified Parkinson’s Disease Rating Scale; MoCA, Montreal Cognitive Assessment; PCs, principal components; PPA, primary progressive aphasia; PPAOS, primary progressive apraxia of speech; PPT, pyramid and palm trees test; PSP, progressive supranuclear palsy; rtvFTD, right temporal variant of frontotemporal dementia; svPPA, semantic variant of primary progressive aphasia; WAB AQ, Western aphasia battery aphasia quotient; WM, white matter.

The MRI, FDG-PET, and tau-PET models all did an excellent job of correctly classifying the PSP participants, AOS-PAA participants, and svPPA participants, with sensitivity between 89% and 100% (Supplementary Figs 1–3, Table 3). The MRI and tau-PET models performed excellently correctly classifying the rtvFTD participants (91% for MRI and 88% for tau-PET), while the FDG-PET model misclassified them as svPPA or AOS-PAA. The FDG-PET model was best able to correctly classify the PPAOS participants with higher sensitivity (82%) than MRI and tau-PET (70%), which misclassified them as PSP or AOS-PAA (Table 3, Supplementary Figs 1–3). All three models had poor-to-moderate sensitivity for bvFTD, CBS, and PPA-other clinical syndromes (Table 3). The tau-PET model was the only model that could correctly classify CBS (70% correctly classified), with participants commonly misclassified as PSP, PPAOS, and AOS-PAA (Supplementary Figs 1–3). The tau-PET and FDG-PET models correctly classified bvFTD more often than the MRI model (58% and 56% correctly classified compared to 25%, respectively), with the models misclassifying participants as PSP, AOS-PAA, and PPAOS (Supplementary Figs 1–3). The models did not have good sensitivity for PPA-other participants: 39% for MRI, 27% for FDG-PET, and 50% for tau-PET (Supplementary Figs 1–3). In cross-validation, all models maintained excellent sensitivity for PSP, while only the MRI and FDG-PET models maintained a good sensitivity for svPPA. The models could not correctly classify the other diagnoses on the testing sets (Table 3). The performance of the model that combined the first 12 PCs from MRI, FDG-PET, and tau-PET was comparable to the performance of models containing all PCs from separate modalities (Tables 2 and 3).

Down-sampling the PSP group substantially improved the sensitivity of the imaging-based models for the least frequent syndromes, including bvFTD and CBS, while decreasing the sensitivity for PSP, except in the tau-PET model (Table 4). Similar trends were obtained with the weighted logistic regression model on MRI-based PCs, whose cross-validated sensitivity and specificity are reported in Supplementary Table 5.

**Table 4:** Sensitivity and specificity of the classifiers on a balanced cohort, down-sampling the PSP group

	Model		Cross-validation	
	Sensitivity	Specificity	Sensitivity	Specificity
MRI PCs				
bvFTD	58.30%	100%	5%	99.70%
svPPA	100%	99.20%	80.30%	97.60%
rtvFTD	81.80%	100%	62.30%	99.20%
AOS-PAA	90%	89.30%	61.90%	75.30%
PPAOS	79.20%	94.80%	39.40%	86.30%
PPA-other	44.40%	100%	13.50%	99.10%
PSP	94.30%	95.30%	75.30%	83.90%
CBS	65.20%	100%	15%	99.30%
FDG-PET PCs				
bvFTD	55.60%	99.50%	23%	99.70%
svPPA	100%	98.40%	87.40%	96.10%
rtvFTD	50%	100%	10%	100%
AOS-PAA	92.70%	90.10%	59.90%	80.90%
PPAOS	82.20%	91.80%	50.60%	81.70%
PPA-other	40%	100%	17%	98.40%
PSP	92.70%	96.90%	80.90%	89.60%
CBS	20%	100%	0%	99.80%
Tau-PET PCs				
bvFTD	91.70%	100%	10%	97.70%
svPPA	100%	98.80%	65.80%	95.60%
rtvFTD	87.50%	100%	43.50%	99%
AOS-PAA	95%	98.60%	48%	80.20%
PPAOS	90%	98%	31.50%	82.40%
PPA-other	87.50%	100%	1.50%	99.40%
PSP	100%	98.60%	67.20%	84.50%
CBS	87%	98.70%	28.60%	92.30%

Sensitivity and specificity of the classifiers were obtained for each syndrome (left), and the predictive capabilities of the classifiers were evaluated with cross-validation (right). For cross-validation, sensitivity and specificity for each diagnosis were calculated as the average value across the testing samples over 100 runs on randomized training and testing splits. Classifiers were built, including all MRI-based principal components (A); all FDG-PET-based principal components (B); all tau-PET-based principal components (C). PCs: principal components. AOS-PAA, apraxia of speech—primary agrammatic aphasia; APOE E4, Apolipoprotein E4; ASRS, Apraxia of speech rating scale; BNT, Boston naming test; bvFTD, behavioural variant of frontotemporal dementia; CBS, corticobasal syndrome; FAB, frontal assessment battery; FDG, fluorodeoxyglucose; GM, grey matter; MDS-UPDRS, Movement Disorder Society-sponsored revision of the Unified Parkinson’s Disease Rating Scale; MoCA, Montreal Cognitive Assessment; PCs, principal components; PPA, primary progressive aphasia; PPAOS, primary progressive apraxia of speech; PPT, pyramid and palm trees test; PSP, progressive supranuclear palsy; rtvFTD, right temporal variant of frontotemporal dementia; svPPA, semantic variant of primary progressive aphasia; WAB AQ, Western aphasia battery aphasia quotient; WM, white matter.

### Additional GLM models

The performance of the imaging-based models only slightly improved when demographics and clinical features were included, including on cross-validation (age, sex, MoCA, MDS-UPDRS III, and FAB) (Table 2). The sensitivity and specificity for the FTD syndromes also increased slightly, with the highest improved sensitivity for PPAOS (Supplementary Table 4).

## Discussion

Using a data-driven unsupervised approach, we found that the FTD clinical syndromes investigated in this study (bvFTD, svPPA, rtvFTD, AOS-PAA, PPAOS, PPA-other, PSP, and CBS) were characterized by both distinct and shared patterns of neuroimaging abnormalities on structural MRI, FDG-PET, and tau-PET. The imaging modes of variations obtained with principal components analysis discriminated between certain syndromes while concurrently finding overlapping patterns of abnormalities, which correlated with selected metrics of clinical impairment. The first FDG-PET and MRI-based principal components yielded similar descriptions of covariance across FTD syndromes, while tau-PET principal components only partly overlapped with them. Logistic regression classifiers built on principal components, including both syndrome-specific and non-specific patterns, performed heterogeneously in discriminating among syndromes. Classifiers’ sensitivity and specificity differed across syndromes, with consistently strong performance for PSP and svPPA and poor performance for CBS and PPA-other. Their performance varied based on the used imaging modality as well as on the inclusion of clinical scores and demographic information.

### Patterns of imaging covariance from principal component analysis

Principal component analysis decomposes the covariance of the original images into orthogonal modes of variation so that each participant’s image can be interpreted as the weighted combination of all the principal components. The principal components are orthogonal, which means that a participant’s/syndrome’s score in one component is not related to their score in another component. Principal components are also ordered in a descending way so that the first one explains the largest amount of variability in the data. For MRI and FDG-PET, the greatest variability (21% for both modalities) corresponded to widespread cortical neurodegeneration, with strong frontal involvement. Participants with more extended cortical atrophy or hypometabolism (i.e. lower PC1 score) were more severely impaired on the MoCA. PC1 MRI was also related to worse scores on several other clinical tests, implying that this component reflected overall severity and encompassed more of the cortex and subcortical structures compared to PC1 FDG-PET. While participants with bvFTD scored low in both MRI and FDG-PET PC1 due to their degree of frontal degeneration, these two components did not generally map well onto any single syndrome. Participants across all syndromes had heterogeneous scores, consistent with the notion that cortical presentation of any FTD syndrome tends to be more aggressive than subcortical presentation, with a faster disease course and worse prognosis.^24^ Somewhat mirroring both PC1 MRI and PC1 FDG-PET, PC1 tau-PET captured a widespread pattern of tau deposition, predominantly in the temporal lobe. PC1 tau-PET explained 43% of the variability within the data, which was almost double the amount of variability explained by PC1 MRI and PC1 FDG-PET, and scores on this PC did not differ across FTD syndromes. This may indicate the lack of specificity of flortaucipir for the 4R and 3R tau inclusions characterizing most FTD syndromes, with PC1 tau-PET reflecting a ‘noise’ component related to off-target binding. Consistent with this hypothesis is the fact that PC1 tau-PET was unrelated to clinical metrics. It appears that the total amount of tau in this PC was more person-specific than syndrome-specific.

The second highest amount of covariance was explained by temporal atrophy, hypometabolism, and tau deposition, in opposition to involvement of brainstem, subcortical, and premotor/motor regions. For all three modalities, PC2 separated svPPA and rtvFTD from the motor and motor speech disorders of PSP, CBS, AOS-PAA, and PPAOS. This is consistent with the striking patterns of anteromedial temporal atrophy, hypometabolism, and uptake on tau-PET in svPPA and rtvFTD.^17,63-66^ The reason why svPPA participants often exhibit elevated flortaucipir binding despite TDP-43, and not tau, being their most common pathology, remains unknown.^66,67^ A similar network of regions can be affected in PSP, CBS, AOS-PAA, and PPAOS, although with varying degrees of involvement of different parts of this network. The midbrain, subthalamic nucleus, thalamus, and cerebellar dentate are typically affected in PSP,^68^ but can also become affected, often to a milder degree, in patients with CBS and AOS-PAA/PPAOS, as they develop clinical features of PSP.^69,70^ Posterior regions of the frontal lobe are commonly affected in CBS, AOS-PAA, and PPAOS^71-74^ and can also be affected in PSP.^68,75^ Another common finding across these syndromes is a relative sparing of the anteromedial temporal lobes. PC2 scores correlated with the degree of parkinsonism, AOS, and the severity of PSP features, pointing to the preservation of temporal structures in the motor syndromes, and, in the opposite direction, with naming and semantic association test scores.

Moving to the subsequent principal components, which explained a smaller fraction of the variability, the imaging modalities appeared increasingly disconnected. PC3 FDG-PET reflected frontal and subcortical hypometabolism, was strongly related to FAB scores and discriminated bvFTD from PPAOS, rtvFTD, and svPPA. Instead, both PC3 and PC4 on MRI reflected atrophy in the brainstem and cerebellum, with PC3 also reflecting atrophy in the occipitoparietal and right temporal lobes, and PC4 in the frontal lobes. The PSP participants showed low scores on both components and could be distinguished from the AOS-PAA, PPAOS, and PPA-other participants, who showed less involvement of brainstem and cerebellar regions. The rtvFTD and CBS participants also showed low scores on PC3, reflecting involvement of the right temporal lobe in rtvFTD and the midbrain in CBS. The component that best identified PPA-other-related abnormalities was PC4 tau-PET, which captured a left frontotemporal pattern that distinguished PPA-other from PPAOS, PSP, CBS, and rtvFTD, none of which typically show aphasia as a predominant feature.

The principal components that we identified were somewhat different from the MRI-based independent components reported in a previous study of FTD syndromes.^14^ This was expected given the differences in the patient’ cohort and data reduction technique (i.e. ICA instead of PCA). However, our findings echo this previous study in the co-existence of multiple clinical syndromes within each atrophy pattern.^14^

### Diagnostic capabilities of principal components

We built classifiers using principal components derived from images, with the advantage of independence from a priori atlas parcellation and warping from template space. The primary goal was to test whether imaging covariance extracted blind to the clinical syndrome was sufficient for a correct diagnosis. Our classifiers trained on imaging-based principal components performed comparably to previously published models trained on regional MRI volumes^34^ or biomarkers^76^ for discriminating among FTD syndromes. The sensitivity and specificity of the models were heterogeneous across syndromes,^34^ although the MRI, FDG-PET, and tau-PET models all performed excellently in correctly classifying PSP and svPPA (>90%). While PSP participants were overrepresented in our cohort (*n* = 182), svPPA participants were not (*n* = 28), with classification accuracy likely so high due to the distinct and focal patterns of neurodegeneration in svPPA.^24^ There is very little overlap in patterns of neurodegeneration between svPPA and PSP. Only 60 of 182 PSP participants had a post-mortem-confirmed diagnosis of PSP. Nevertheless, the imaging-based classifiers were able to discriminate PSP from the other syndromes, even in the testing sets of the cross-validation process, potentially thanks to the contribution of PC2 of all three imaging modalities. All three models also did an excellent job in correctly classifying AOS-PAA participants (>85%).

Model performance in the remaining syndromes varied by modality. The FDG-PET model was best able to correctly classify the PPAOS participants, with a sensitivity of 82% and a specificity of 96%. The MRI and tau-PET models had a sensitivity of 70% for PPAOS, misclassifying these participants as PSP or AOS-PAA. Indeed, the voxel-level maps showed greater severity and topographic extent of abnormalities in the posterior frontal lobes in PPAOS on FDG-PET compared to the other two modalities. In contrast, the MRI and tau-PET models performed better than FDG-PET in classifying the rtvFTD participants. FDG-PET had a sensitivity of only 25% for rtvFTD, with participants misclassified as svPPA or AOS-PAA. This low sensitivity in detecting rtvFTD was likely due to the very limited number of participants diagnosed with rtvFTD who underwent FDG-PET (*n* = 4).

Relative to MRI, FDG-PET and tau-PET best classified the bvFTD participants, although only 56% and 58%, respectively. Instead, only the tau-PET classifier had good sensitivity for CBS (70%). This finding is unexpected because tau-PET showed very little uptake in both bvFTD and CBS. The absence of elevated flortaucipir binding may be related to the majority of bvFTD and CBS participants being A*β*-negative, given that amyloid positivity is the strongest predictor of tau positivity in FTD syndromes.^65,77^ This suggests that it may have been the absence of tau-PET signal that helped to differentiate these syndromes from the other syndromes that showed stronger patterns of uptake. The MRI and FDG-PET models frequently misclassified both bvFTD and CBS participants as PSP, AOS-PAA, and PPAOS. Our voxel-level maps showed frontal and basal ganglia atrophy and hypometabolism in both bvFTD and CBS, albeit with more prefrontal involvement in bvFTD and premotor/motor involvement in CBS. These patterns are typical for those previously associated with these syndromes.^72,76,78^ Involvement of the frontal lobes in PSP, AOS-PAA, and PPAOS might have led to misclassification. The presence of the PSP-CBS clinical subtype in the PSP group, as well as other cortical subtypes of PSP, might also have posed challenges in correctly separating CBS participants from PSP.^79^ Furthermore, CBS and bvFTD are pathologically heterogeneous.^79-81^ Only three CBS participants had a pathological diagnosis in our cohort, and CBD, PSP, and LBD pathologies were observed. Conversely, CBD pathology was observed in participants with bvFTD, PSP, PPA-other, AOS-PAA, and PPAOS, increasing the potential for overlap and misclassification. A previous study reported that covariance patterns extracted from FDG-PET relative to healthy controls can achieve high sensitivity in separating bvFTD from Alzheimer’s disease, svPPA, and nfvPPA.^36^ We purposely did not include cognitively unimpaired participants in the principal component analysis since our overarching goal was to identify image covariance within the FTD syndromes, more than to identify the phenotypical characteristics that are most diagnostically useful. The diagnostic utility obtained may also differ depending on the mix of comparison syndromes.

None of the three modalities were able to correctly classify the PPA-other participants, possibly because this group was heterogeneous and showed overlap with several of the other syndromes.^82^ PPA-other participants also had shorter disease duration than any other group, which might have been detrimental for the classifiers, rendering some participants’ features less mature and recognizable. Additionally, the eight PPA-other participants who underwent autopsy had heterogeneous pathological diagnoses, including CBD, TDP-43, PiD, GGT, and ADNC. ADNC at autopsy was found in one logopenic PPA participant, who was A*β*-negative at the visit included in the current study, meeting inclusion criteria.

The performance of the models was poorer after cross-validation, as expected, and good sensitivity was retained only for PSP and svPPA. Despite flortaucipir not being optimized for 3R and 4R tau, which are the pathological hallmark of FTD tauopathies, models based on tau-PET principal components had a higher AUROC than MRI and FDG-PET models. However, tau-PET principal components performed less well on unseen cases in cross-validation, with the AUROC dropping from 0.88 to 0.71, implying that the model trained on the entire dataset might have been overfit. The best diagnostic performance on the unseen cases in cross-validation was achieved by FDG-PET principal components. MRI-based principal components performed similarly to FDG-PET in cross-validation, implying that patterns of atrophy and hypometabolism have comparable diagnostic potential. The model that combined the first 12 PCs from MRI, FDG-PET, and tau-PET did not substantially improve the AUROC values over the separate modality models. This could be expected when diagnostic performance for each syndrome differs across modalities. It may also suggest that the variance explained by subsequent components included in the single modality models, despite being small, were crucial in discriminating between these syndromes. However, this model showed the highest sensitivity for bvFTD participants on cross-validation (29%).

Adding clinical and demographic scores to the image-based classifiers modestly improved their diagnostic performance, suggesting the utility of combined clinical and imaging models to aid diagnosis. However, we hypothesize that the classifiers would have achieved higher accuracy if more syndrome-specific clinical scores were added as predictors. The addition of clinical and demographic scores dramatically improved the sensitivity for PPAOS, particularly after cross-validation. We attribute this to the contribution of MoCA, where PPAOS performed significantly better than participants with other FTD syndromes, including AOS-PAA and PSP.

The performance of the logistic regression classifiers must be interpreted in the context of an imbalanced cohort, skewed towards PSP, which is the main limitation of our study. After down-sampling the PSP group, the sensitivity of the imaging-based models for the least-frequent syndromes largely improved. However, the sensitivity remained the highest for svPPA and PSP.

## Conclusions

The strengths of our study are the multimodal images available for a large cohort of participants clinically diagnosed with eight different FTD syndromes and the use of *w*-scored images, which allowed us to investigate variability purely within the disease, excluding the confounding factors of normal ageing and sex. The limitations of our study include a cohort skewed towards PSP, PPAOS, and AOS-PAA, with only 12 bvFTD participants, due to the nature of the grants supporting our research, and absence of autopsy-confirmed diagnosis for most participants. Additionally, not all clinical scores were homogeneously available across syndromes, and this limited the inclusion of additional tests in the classifiers. To identify patterns of imaging covariance and test their diagnostic abilities, we employed principal component analysis and logistic regression models. Future studies may investigate other techniques for data reduction, such as NNMF,^40^ and more sophisticated machine learning models for syndrome classification. Future directions of our work also include the characterization of the covariance in longitudinal rates of change of imaging abnormalities and in imaging modalities not included in the present work, such as diffusion tensor imaging for white matter tracts disruption, functional MRI, and tau-PET with second-generation tracers.

In summary, our study found that the covariance components of structural and molecular neuroimaging underlying FTD clinical syndromes exist as a continuum and with syndrome-specific patterns. These covariance imaging patterns alone were capable of accurately discriminating among some syndromes but not others, and the contribution of clinical scores as well as the combination of patterns from different modalities improved such capabilities, underscoring the aid of neuroimaging in establishing patient diagnosis.

## Supplementary Material

fcag341_Supplementary_Data

## Data Availability

Data and scripts (R, MATLAB) that support the findings of this study are available from the corresponding author upon reasonable request.
