# Supplementary material for: Distinct and shared multimodal neuroimaging patterns in clinical frontotemporal dementia syndromes
Source: Brain Commun. 2026 Sep 10;8(5):fcag341. doi: 10.1093/braincomms/fcag341 (PMC13583894; doi:10.1093/braincomms/fcag341)

Supplementary Table 1. PSP clinical variants. PSP clinical variants included Richardson' syndrome (PSP-RS), speech and language (PSP-SL), oculomotor (PSP-OM), parkinsonism (PSP-P), frontal (PSP-F), progressive gait freezing (PSP-PGF), corticobasal syndrome (PSP-CBS), postural instability (PSP-PS), and primary lateral sclerosis (PSP-PLS).

**Clinical PSP variants**	**N**
**PSP-RS**	94
**PSP-subcortical**	
PSP-P	30
PSP-PGF	14
PSP-OM	1
PSP-PI	6
PSP-PLS	7
**PSP-cortical**	
PSP-CBS	18
PSP-SL	7
PSP-F	8

Supplementary Table 2. Pathological diagnoses**.** TDP-43: Tar DNA-binding protein 43. MSA: Multiple system atrophy. MELAS: Mitochondrial encephalopathy with lactic-acidosis and stroke-like episodes syndrome. LBD: Lewy Body disease. PiD: Pick's disease. GGT: Globular glial tauopathy. ADNC: Alzheimer's disease neuropathological changes. DNTC: diffuse neurofibrillary tangles with calcification.

	bvFTD	***svPPA***	***rtvFTD***	***AOS-PAA***	***PPAOS***	***PPA-other***	***PSP***	***CBS***
Number of participants with autopsy	4	4	5	23	20	8	60	3
CBD	2	0	0	11	7	1	5	1
TDP 43	2	3	4	3	0	3	2	0
PiD	0	1	0	2	1	2	0	0
PSP	0	0	0	7	11	0	45	1
LBD	0	0	0	0	0	0	1	1
GGT	0	0	1	0	0	1	1	0
ADNC	0	0	0	0	0	1	0	0
Mixed 3R and 4R frontotemporal tauopathy	0	0	0	0	1	0	0	0
MSA	0	0	0	0	0	0	4	0
MELAS	0	0	0	0	0	0	1	0
DNTC	0	0	0	0	0	0	1	0

Supplementary Table 3. Number and percent of participants for each diagnosis who underwent each clinical test. bvFTD: behavioral variant of frontotemporal dementia. svPPA: semantic variant of primary progressive aphasia. rtvFTD: right temporal variant of frontotemporal dementia. AOS-PAA: Apraxia of speech - primary agrammatic aphasia. PPAOS: Primary progressive apraxia of speech. PPA: primary progressive aphasia. PSP: progressive supranuclear palsy. CBS: Corticobasal syndrome. APOE E4: Apolipoprotein E4. MoCA: Montreal Cognitive Assessment. MDS-UPDRS: Movement Disorder Society-sponsored revision of the Unified Parkinson's Disease Rating Scale. ASRS: Apraxia of speech rating scale. FAB: Frontal assessment battery. BNT: Boston Naming Test. WAB AQ: Western PPA battery PPA quotient. PPT: Pyramids and Palm Trees Test.

	**bvFTD**	**svPPA**	**rtvFTD**	**AOS-PAA**	**PPAOS**	**PPA**	**PSP**	**CBS**
**MoCA**	n=11, 92%	n=27, 96%	n=10, 91%	n=69, 99%	n=53, 100%	n=17, 94%	n=181, 98%	n=22, 96%
**MDS-UPDRS III**	n=11, 92%	n=28, 100%	n=11, 100%	n=69, 99%	n=52, 98%	n=18, 100%	n=183, 99%	n=20, 87%
**PSP Rating Scale**	n=11, 92%	n=19, 68%	n=9, 82%	n=47, 67%	n=37, 70%	n=8, 44%	n=183, 99%	n=19, 83%
**ASRS Total v3**	n=10, 83%	n=27, 96%	n=11, 100%	n=61, 87%	n=51, 96%	n=16, 89%	n=134, 72%	n=13, 57%
**FAB**	n=12, 100%	n=28, 100%	n=11, 100%	n=66, 94%	n=51, 96%	n=17, 94%	n=178, 96%	n=19, 83%
**BNT**	n=5, 42%	n=11, 39%	n=2, 18%	n=63, 90%	n=49, 92%	n=15, 83%	n=130, 70%	n=16, 70%
**Letter fluency sum**	n=9, 75%	n=26, 93%	n=9, 82%	n=61, 87%	n=40, 75%	n=14, 78%	n=32, 17%	n=11, 48%
**WAB praxis**	n=10, 83%	n=28, 100%	n=11, 100%	n=69, 99%	n=52, 98%	n=16, 89%	n=7, 4%	n=15, 65%
**WAB AQ**	n=9, 75%	n=27, 96%	n=11, 100%	n=62, 89%	n=52, 98%	n=17, 94%	n=66, 36%	n=12, 52%
**WAB repetition**	n=9, 75%	n=27, 96%	n=11, 100%	n=63, 90%	n=50, 94%	n=17, 94%	n=36, 19%	n=11, 48%
**WAB animal fluency**	n=9, 75%	n=27, 96%	n=11, 100%	n=63, 90%	n=50, 94%	n=17, 94%	n=35, 19%	n=14, 61%
**PPT word-word**	n=6, 50%	n=27, 96%	n=10, 91%	n=41, 59%	n=30, 57%	n=17, 94%	n=1, 1%	n=1, 4%

Supplementary Table 4. Sensitivity and specificity of the classifiers for each syndrome. Sensitivity and specificity of the classifiers were obtained for each syndrome (left), and the predictive capabilities of the classifiers were evaluated with cross-validation (right). For cross-validation, sensitivity and specificity for each diagnosis were calculated as the average value across the testing samples over 100 runs on randomized training and testing splits. Classifiers were built including: demographics (age, sex), clinical scores (MoCA, MDS-UPDRS III, FAB), and all principal components from MRI (A), or FDG-PET (B), or tau-PET (C); first 12 principal components from MRI, FDG-PET and tau-PET (D).

	Model		*Cross-validation*	
	Sensitivity	***Specificity***	***Sensitivity***	***Specificity***
MRI PCs, clinical, demographics				
AOS-PAA	78.30%	96.80%	49.60%	90.10%
bvFTD	66.70%	100%	3.30%	100%
CBS	50%	100%	3.50%	100%
PPA-other	66.70%	100%	3%	99.70%
PPAOS	80%	95.60%	53.80%	92.90%
PSP	98%	91.40%	95.10%	72%
rtvFTD	66.70%	100%	52.50%	99.60%
svPPA	100%	98.80%	86.80%	97.60%
FDG-PET PCs, clinical, demographics				
AOS-PAA	94%	97%	57%	89.20%
bvFTD	66.70%	100%	14%	100%
CBS	0%	100%	0%	100%
PPA-other	50%	100%	8%	99.10%
PPAOS	90.70%	96.20%	55.90%	88.80%
PSP	99%	92.70%	92.90%	80.20%
rtvFTD	75%	100%	7%	100%
svPPA	95.50%	98.70%	88.20%	97.10%
Tau-PET PCs, clinical, demographics				
AOS-PAA	97.20%	97.80%	38.80%	92.60%
bvFTD	72.70%	100%	12.30%	99.60%
CBS	68.40%	100%	1.80%	99.50%
PPA-other	28.60%	100%	1%	100%
PPAOS	85.70%	98.70%	45.20%	92.70%
PSP	99.20%	90.50%	96.20%	62.40%
rtvFTD	87.50%	100%	27%	99.40%
svPPA	94.10%	99.20%	65.20%	96.70%
MRI, FDG-PET, Tau-PET (first 12 PCs), clinical, demographics				
AOS-PAA	78.30%	96.80%	45.20%	93.20%
bvFTD	66.70%	100%	40%	98.50%
CBS	50%	100%	0.50%	99.80%
PPA-other	66.70%	100%	3.50%	99.80%
PPAOS	80%	95.60%	61%	91.80%
PSP	98%	91.40%	96.80%	77.30%
rtvFTD	66.70%	100%	-	-
svPPA	100%	98.80%	92.70%	98.70%

Supplementary Table 5. Sensitivity and specificity of the MRI-based weighted classifier. Comparison of the sensitivities and specificities of the MRI-based logistic regression models with (left) and without (right) weights, after cross-validation.

	Model with weights		***Model without weights***	
	Sensitivity	***Specificity***	***Sensitivity***	***Specificity***
MRI PCs				
bvFTD	8.70%	98.20%	1%	100%
svPPA	82.50%	98%	80%	97.60%
rtvFTD	85.30%	99%	51.70%	99.60%
AOS-PAA	41.60%	86.70%	44.50%	89.60%
PPAOS	33%	86.40%	20.10%	95%
PPA-other	10%	97.60%	2%	99.70%
PSP	74.70%	81.60%	93.60%	56.20%
CBS	20.20%	95.30%	0.40%	100%

Supplementary Figure 1. Classifier predictions. The predictions of the MRI-based classifier were plotted for each syndrome: bvFTD (**A**), svPPA (**B**), rtvFTD (**C**), AOS-PAA (**D**), PPAOS (**E**), PPA-other (**F**), PSP (**G**), CBS (**H**). The classifier was built with all MRI-based principal components (n=400) The number and percentage of participants classified to each diagnosis is reported above each histogram column. bvFTD: behavioral variant of frontotemporal dementia. svPPA: semantic variant of primary progressive aphasia. rtvFTD: right temporal variant of frontotemporal dementia. AOS-PAA: Apraxia of speech - primary agrammatic aphasia. PPAOS: Primary progressive apraxia of speech. PPA: primary progressive aphasia. PSP: progressive supranuclear palsy. CBS: Corticobasal syndrome.

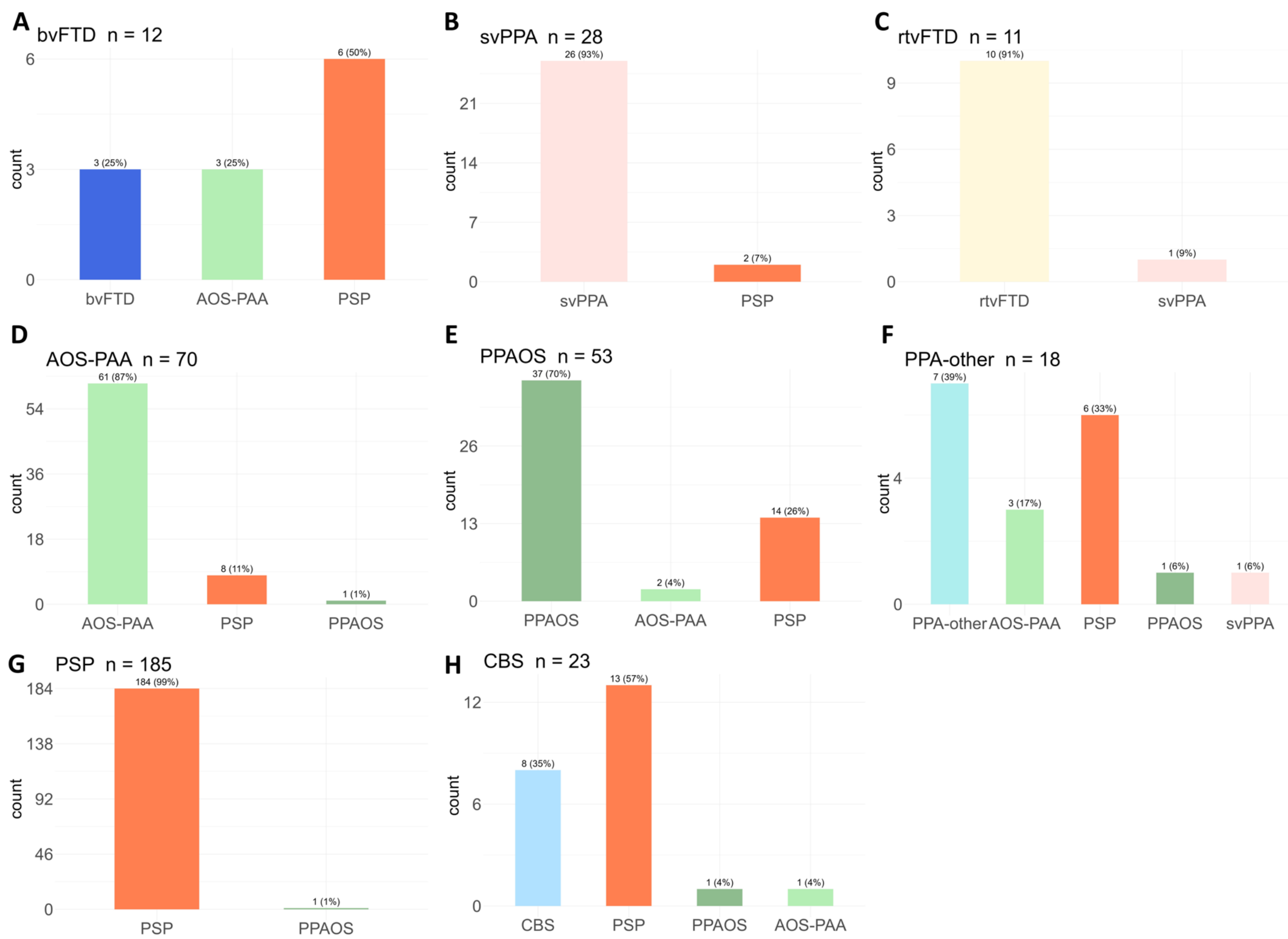

Supplementary Figure 2. Classifier predictions. The predictions of the FDG-PET-based classifier were plotted for each syndrome: bvFTD (**A**), svPPA (**B**), rtvFTD (**C**), AOS-PAA (**D**), PPAOS (**E**), PPA-other (**F**), PSP (**G**), CBS (**H**). The classifier was built with all FDG-PET-based principal components (n=267). The number and percentage of participants classified to each diagnosis is reported above each histogram column. bvFTD: behavioral variant of frontotemporal dementia. svPPA: semantic variant of primary progressive aphasia. rtvFTD: right temporal variant of frontotemporal dementia. AOS-PAA: Apraxia of speech - primary agrammatic aphasia. PPAOS: Primary progressive apraxia of speech. PPA: primary progressive aphasia. PSP: progressive supranuclear palsy. CBS: Corticobasal syndrome.

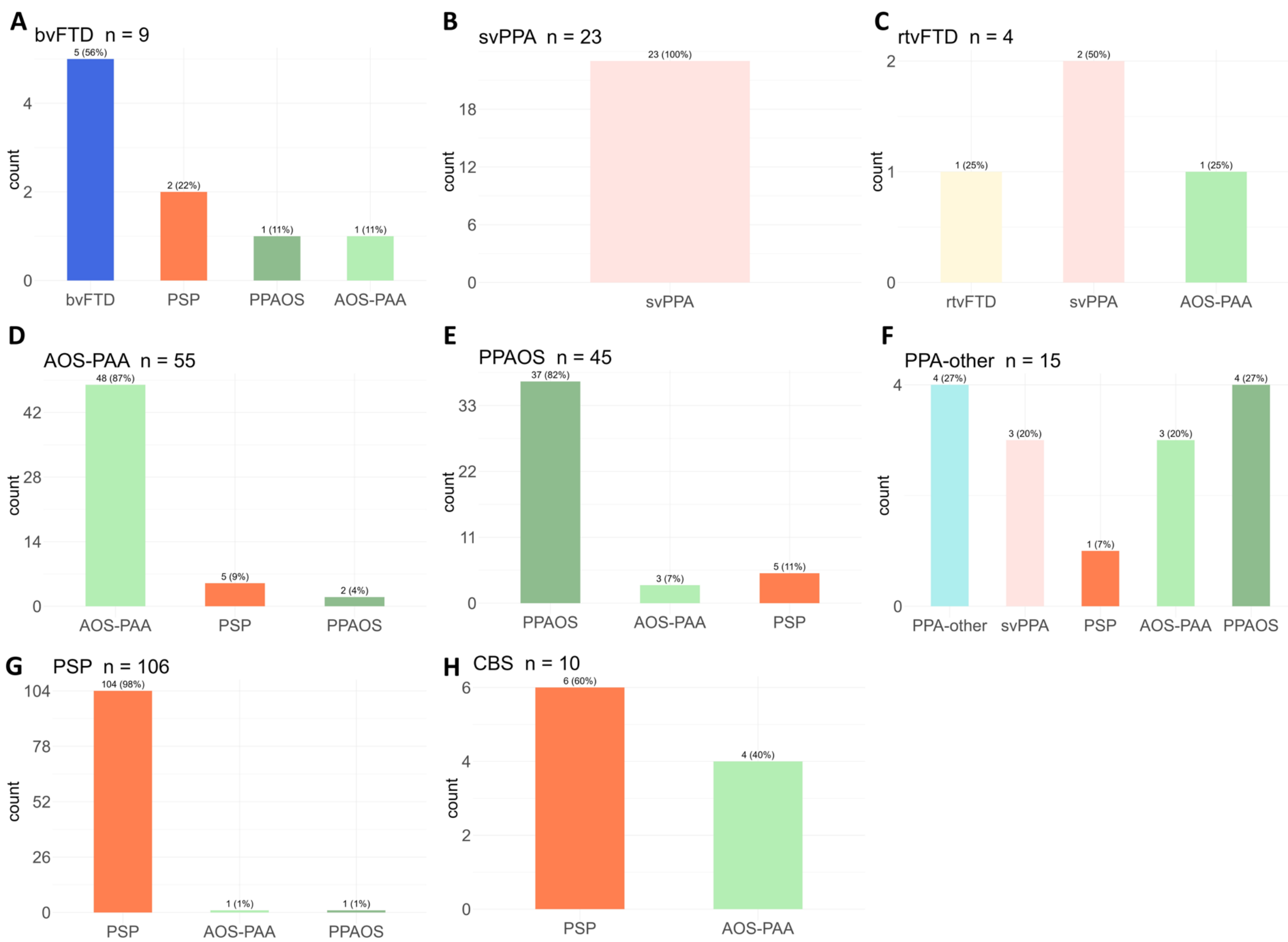

Supplementary Figure 3. Classifier predictions. The predictions of the tau-PET-based classifier were plotted for each syndrome: bvFTD (**A**), svPPA (**B**), rtvFTD (**C**), AOS-PAA (**D**), PPAOS (**E**), PPA-other (**F**), PSP (**G**), CBS (**H**). The classifier was built with all tau-PET-based principal components (n=279). The number and percentage of participants classified to each diagnosis is reported above each histogram column. bvFTD: behavioral variant of frontotemporal dementia. svPPA: semantic variant of primary progressive aphasia. rtvFTD: right temporal variant of frontotemporal dementia. AOS-PAA: Apraxia of speech - primary agrammatic aphasia. PPAOS: Primary progressive apraxia of speech. PPA: primary progressive aphasia. PSP: progressive supranuclear palsy. CBS: Corticobasal syndrome.

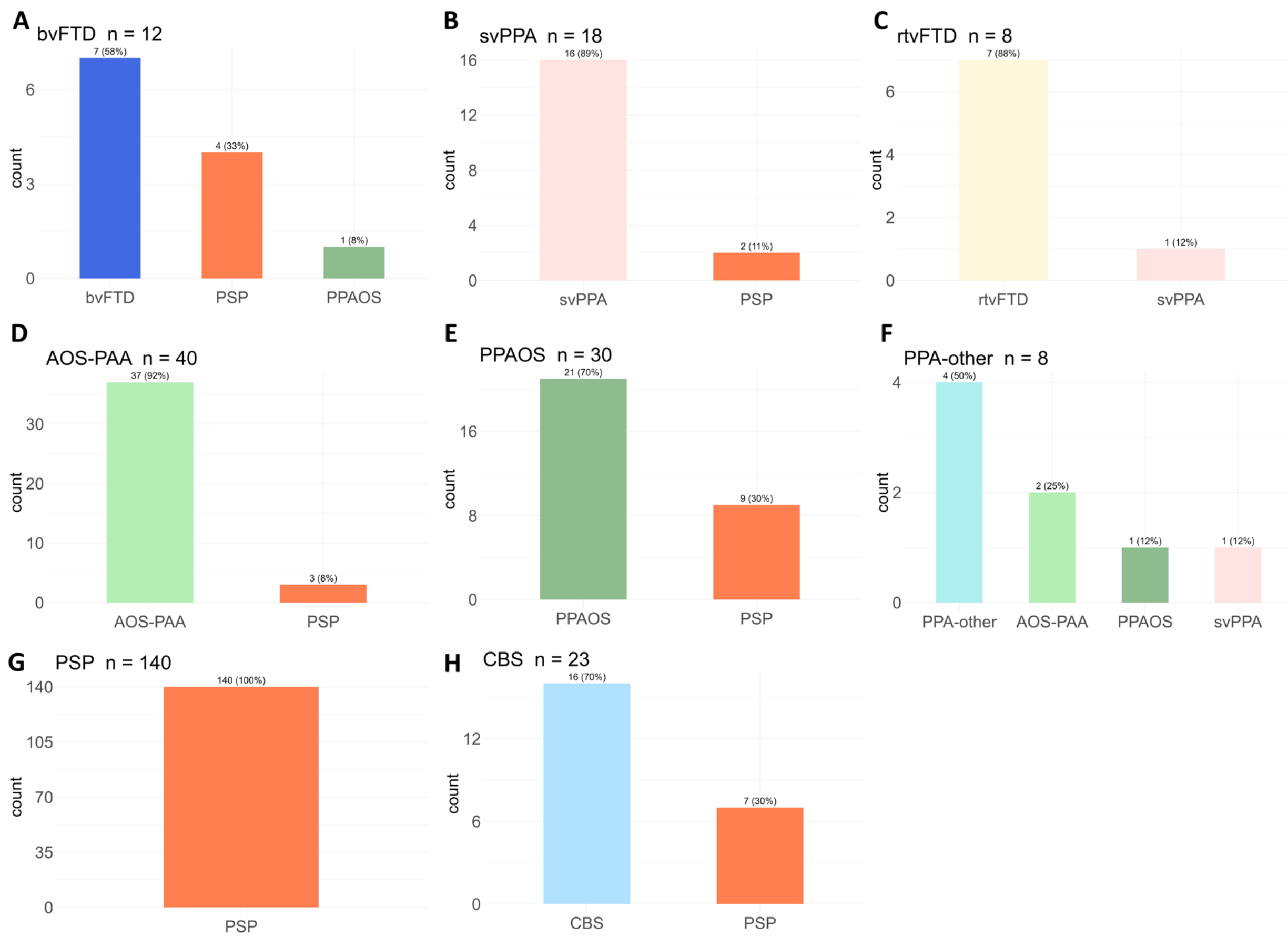

Supplement: Supplementary file 1 [file fcag341_supplementary_data.pdf]
